# Analysis of the treatment efficacy and prognostic factors of PD-1/PD-L1 inhibitors for advanced gastric or gastroesophageal junction cancer: a multicenter, retrospective clinical study

**DOI:** 10.3389/fimmu.2024.1468342

**Published:** 2024-10-24

**Authors:** Yuanyuan Yang, Zhe Wang, Dao Xin, Lulu Guan, Bingtong Yue, Qifan Zhang, Feng Wang

**Affiliations:** ^1^ Department of Medical Oncology, National Cancer Center/National Clinical Research Center for Cancer/Cancer Hospital, Chinese Academy of Medical Sciences and Peking Union Medical College, Beijing, China; ^2^ Department of Oncology, The First Affiliated Hospital of Zhengzhou University, Zhengzhou, China; ^3^ Department of Clinical Medicine, The First Clinical Medical College, Zhengzhou University, Zhengzhou, China; ^4^ Henan Key Laboratory of Chronic Disease Prevention and Therapy & Intelligent Health Management, Zhengzhou, China

**Keywords:** PD-1/PD-L1 inhibitors, gastric or gastroesophageal junction cancer, predictive biomarkers, prognosis, efficacy

## Abstract

**Introduction:**

Immune checkpoint inhibitors (ICIs) have transformed advanced gastric cancer treatment, yet patient responses vary, highlighting the need for effective biomarkers. Common markers, such as programmed cell death ligand-1 (PD-L1), microsatellite instability/mismatch repair (MSI/MMR), tumor mutational burden, tumor-infiltrating lymphocytes, and Epstein–Barr virus, face sampling challenges and high costs. This study seeks practical, minimally invasive biomarkers to enhance patient selection and improve outcomes.

**Methods:**

This multicenter retrospective study analyzed 617 patients with advanced gastric or gastroesophageal junction cancer treated with programmed cell death protein-1 (PD-1)/PD-L1 inhibitors from January 2019 to March 2023. Clinical data and peripheral blood marker data were collected before and after treatment. The primary endpoints were overall survival (OS) and progression-free survival (PFS); the secondary endpoints included the objective response rate (ORR) and disease control rate (DCR). Least absolute shrinkage and selection operator (LASSO)-Cox and LASSO logistic regression analyses identified independent factors for OS, PFS, and ORR. Predictive nomograms were validated using receiver operating characteristic (ROC) curves, areas under the curve (AUCs), C-indices, and calibration curves, with clinical utility assessed via decision curve analysis (DCA), net reclassification improvement (NRI), and integrated discrimination improvement (IDI).

**Results:**

OS-related factors included treatment line, T stage, ascites, pretreatment indirect bilirubin (pre-IBIL), posttreatment CA125, CA199, CA724, and the PLR. PFS-related factors included treatment lines, T stage, metastatic sites, pre-IBIL, posttreatment globulin (GLOB), CA125, and CA199 changes. ORR-related factors included treatment line, T stage, N stage, liver metastasis, pretreatment red cell distribution width-to-platelet ratio (RPR), CA125, and CA724 changes. The nomograms showed strong predictive performance and clinical utility.

**Conclusions:**

Early treatment, lower T stage, the absence of ascites, and lower pre-IBIL, post-CA125, CA199, CA724, and PLR correlate with better OS. Factors for improved PFS include early treatment, lower T stage, fewer metastatic sites, and lower pre-IBIL, post-GLOB, and post-CA125 levels. Nomogram models can help identify patients who may benefit from immunotherapy, providing valuable clinical guidance.

## 1 Introduction

Gastric cancer is one of the most common malignancies, ranking fifth in incidence and fourth in mortality globally in 2020 (1). It has a poor prognosis, with a global five-year survival rate between 20% and 40% (2). During the chemotherapy era, treatments for advanced gastric cancer include fluoropyrimidines and platinum or paclitaxel-based regimens, resulting in a survival time of approximately one year (3, 4). Immune checkpoint inhibitors (ICIs) have significantly improved survival in advanced gastric cancer patients, as shown in large phase III trials, such as the CheckMate 649, ATTRACTION-4, KEYNOTE-859, and KEYNOTE-811 studies. However, responses to ICIs vary, even among patients with programmed cell death ligand-1 (PD-L1) positivity or microsatellite instability (MSI-H) status. Some PD-L1-negative or microsatellite-stable patients may benefit from ICIs (5–7). Therefore, it is crucial to identify simple, accurate, and accessible biomarkers to predict which gastric cancer patients might benefit from immunotherapy.

Current clinical prognostic assessments, including assessments of tumor infiltration depth, lymph node metastasis, hematogenous metastasis, tumor location, histological grade, and lymphovascular invasion, are based on the American Joint Committee on Cancer (AJCC) staging system (8, 9). However, factors such as age, sex, tumor differentiation, and immunotherapy cycles, which may be significant for individual survival prediction, were not fully accounted for. Common biomarkers include PD-L1 expression, MSI/mismatch repair status, tumor mutational burden, and circulating tumor DNA, but some potential biomarkers, such as peripheral blood inflammation markers, tumor markers, and nutritional status, remain controversial.

The inflammatory response in the tumor microenvironment is closely related to tumor occurrence, progression, invasion, and metastasis (10). Peripheral blood inflammatory markers can not only predict gastric cancer prognosis (11–17) but are also linked to immunotherapy responses (18–21). Baseline serum tumor marker concentrations and their dynamic changes can also predict ICI outcomes (22–25). Huang J et al. reported that the serum levels of carcinoembryonic antigen (CEA) and CA125 predict progression-free survival (PFS) and overall survival (OS) in patients with non-small cell lung cancer (NSCLC) receiving first-line immunotherapy (26). Additionally, nutritional status is important for gastric cancer patients due to the anatomical features of the stomach (27–30). Albumin, prealbumin, and body mass index (BMI) are independent prognostic factors for gastric cancer (31). A low prognostic nutritional index (PNI) score before treatment was proven to be an independent risk factor for survival in advanced NSCLC patients receiving programmed cell death protein-1 (PD-1) inhibitors (32–34).

This study aimed to evaluate comprehensive clinical and pathological data, including peripheral blood inflammatory markers, tumor markers, and nutritional indices, to identify predictive biomarkers for advanced gastric cancer patients treated with PD-1/PD-L1 inhibitors. We hope to develop a robust prognostic model that enhances treatment precision and offers personalized clinical guidance. By integrating diverse biomarkers, we aim to improve patient outcomes and optimize the use of immunotherapy, ultimately refining therapeutic decision-making in advanced gastric cancer patients.

## 2 Methods

### 2.1 Study population

This retrospective study included 617 patients with advanced gastric or gastroesophageal junction cancer who received ICI treatment from January 2019 to March 2023 at The First Affiliated Hospital of Zhengzhou University, Henan Cancer Hospital, and Anyang Cancer Hospital.

The inclusion criteria for patients were as follows: (1) were over 18 years of age with histologically or cytologically confirmed gastric or gastroesophageal junction cancer; (2) had locally advanced unresectable, recurrent, or metastatic disease; (3) had undergone at least two cycles of systemic treatment based on PD-1/PD-L1 inhibitors; (4) had an Eastern Cooperative Oncology Group performance status of 0-2; (5) had at least one measurable target lesion that could be monitored by computed tomography or magnetic resonance imaging; (6) had normal vital organ function; (7) had complete clinical data, including routine blood, liver and kidney function data and tumor marker data, one week before treatment and after two treatment cycles, before PD-1/PD-L1 inhibitor treatment; and (8) had regularly scheduled follow-up data available.

The exclusion criteria were as follows: (1) patients with other primary malignancies; (2) patients without assessable lesions or who did not undergo regular efficacy evaluations; (3) patients who experienced relapse within six months after neoadjuvant or adjuvant therapy; (4) patients with a history of surgery within the last month; (5) patients with severe infections or inflammatory diseases prior to immunotherapy; (6) patients with serious heart, cerebrovascular, lung, liver, or kidney diseases or other major illnesses that would prevent tolerance to treatment; (7) patients with autoimmune diseases or other immune system deficiencies; (8) patients who were using or had a long-term history of using hematopoietic factors, hormones, or immunosuppressive drugs; (9) patients allergic to PD-1/PD-L1 inhibitors or those with metabolic disorders; (10) patients with psychiatric disorders, a history of substance abuse, or who could not discontinue such substances; and (11) pregnant or breastfeeding women.

The primary endpoints were OS and PFS, while the secondary endpoints included the objective response rate (ORR) and disease control rate (DCR). OS was defined as the time from the start of PD-1/PD-L1 inhibitor treatment to death from any cause or the last follow-up, and PFS was defined as the time from the start of treatment to the first occurrence of disease progression, death, or last follow-up. The ORR was defined as the proportion of patients who achieved complete response (CR) or partial response (PR), and the DCR was defined as the proportion of patients who achieved CR, PR, or stable disease (SD). Patient efficacy was evaluated according to the Response Evaluation Criteria in Solid Tumors version 1.1. All patients were followed up regularly after the initiation of treatment to monitor disease recurrence or progression. The final follow-up date was August 31, 2023.

This study complied with the principles of the Helsinki Declaration and relevant ethical requirements and was approved by the Ethics Committee of Scientific Research and Clinical Trials of the First Affiliated Hospital of Zhengzhou University (Approval Identifier: 2023-KY-1308-002).

### 2.2 Study variables

We collected the pretreatment indicators of gastric cancer patients who met the inclusion criteria as follows. The patients’ clinicopathological characteristics included sex, age, smoking history, alcohol consumption history, BMI, PD-L1 combined positive score (CPS), human epidermal growth factor receptor 2 (Her-2) expression, Ki-67 expression, pathological type, differentiation degree, and Lauren classification.

The tumor characteristics included the primary tumor location, TNM stage, sites of metastasis (e.g., liver, bone, lymph nodes, lung, peritoneum, malignant ascites), and number of metastatic sites. Treatment details included drug names, treatment regimens, treatment lines, presence of radical surgery, radiotherapy, start time of treatment, and progression time.

Hematological data included hemoglobin (Hb), platelet (PLT) count, neutrophil (Neut) count, lymphocyte (Lym) count, monocyte (Mono) count, red cell distribution width (RDW), total protein (TP), albumin (ALB), globulin (GLOB), total bilirubin (TBIL), direct bilirubin (DBIL), IBIL, CA125, CA199, CA724, and CEA. Moreover, hematological indicators were collected not only at baseline but also after two cycles of treatment.

Additionally, we calculated comprehensive indices before the first treatment and after two treatment cycles: PNI = ALB + 5 × Lym, the neutrophil-to-Lym ratio (NLR) = Neut/Lym, the platelet-to-lymphocyte ratio (PLR) = PLT/Lym, the monocyte-to-lymphocyte ratio (MLR) = Mono/Lym, the neutrophil-to-monocyte ratio (NMR) = Neut/Mono, the systemic immune-inflammation index (SII) = PLT× Neut/Lym, the neutrophil-to-lymphocyte ratio (NLPR) = Neut/Lym×PLT, the aggregate index of systemic inflammation (AISI) = Neut×PLT×Mono/Lym, the systemic inflammation response index (SIRI)= Neut×Mono/Lym, the red cell distribution width-to-albumin ratio (RAR)=RDW/ALB, the red cell distribution width-to-platelet ratio (RPR) = RDW/PLT, the red cell distribution width-to-lymphocyte ratio (RLR) = RDW/Lym, and the hemoglobin-to-platelet ratio (HPR) = Hb/PLT. Changes in tumor markers and comprehensive indices were calculated by subtracting pretreatment values from posttreatment values.

### 2.3 Study design and statistical analysis

**Figure 1:**
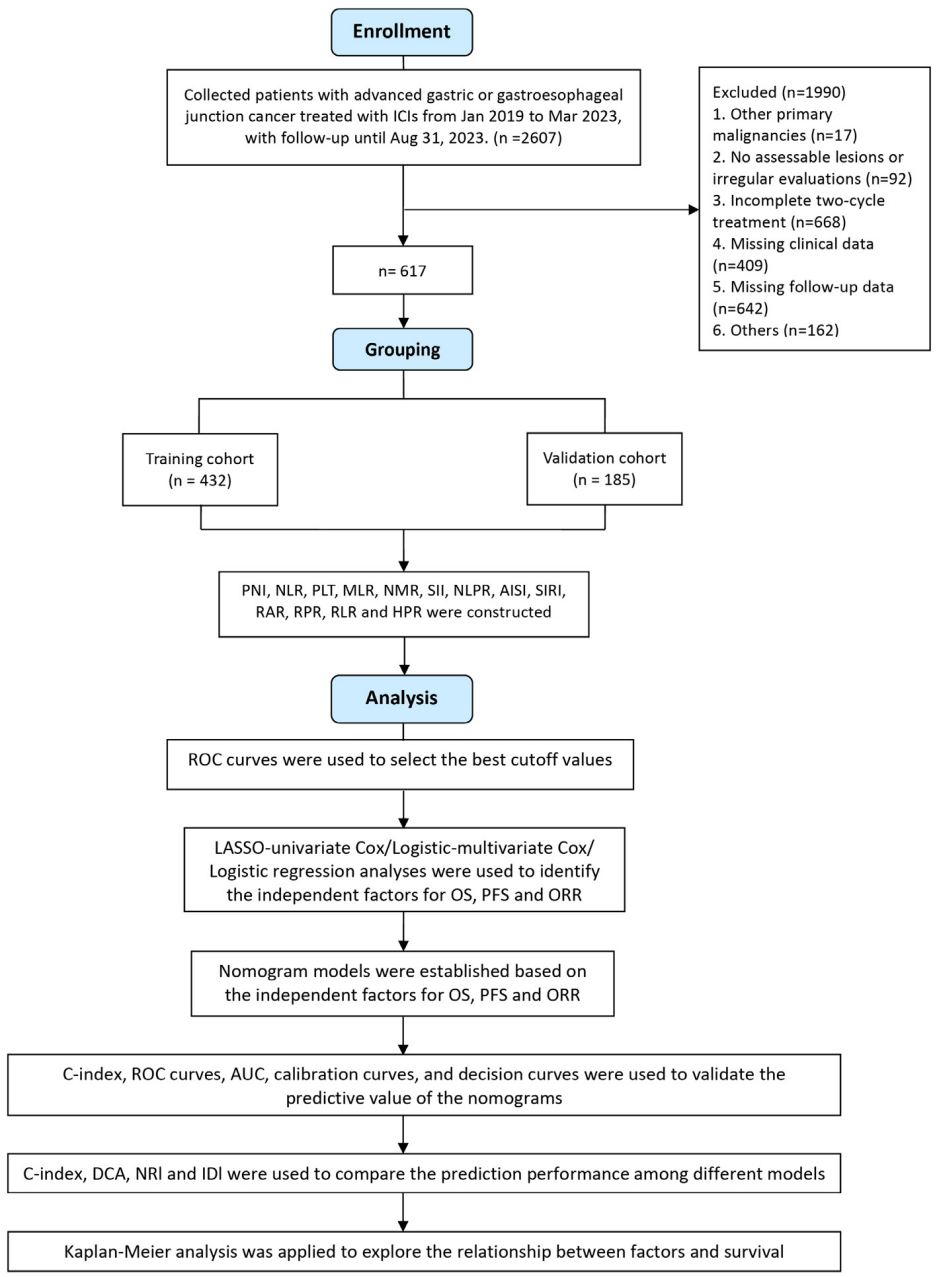
Flow chart of the study design.

The study design is shown in **Figure 1**. This study focused on patient survival status as the outcome variable. Receiver operating characteristic (ROC) curves were used to calculate the Youden index, and the corresponding level of each indicator at which the Youden index was maximized was taken as the optimal cutoff value. If the Youden index was not available or there was a significant difference in group size, the median was used as the cutoff. The upper limit of normal values was used as the cutoff of tumor markers. Patients were divided into high and low groups based on these values, and changes in indicators were categorized by whether values increased or decreased after immunotherapy.

All patients were randomly assigned to training and validation cohorts at a 7:3 ratio, and the χ2 test was applied to compare the intergroup differences. LASSO-Cox regression identified independent predictors for OS and PFS, while LASSO logistic regression identified predictors for ORR. These predictors were used to construct nomogram models for OS, PFS, and ORR. The model’s discriminative ability was assessed using ROC curves and area under the curve (AUC), and the C-index and calibration ability were evaluated using calibration plots. The net benefit of the nomogram in a clinical setting was assessed by decision curve analysis (DCA). To further evaluate the clinical benefit and utility of the nomogram model compared to the AJCC staging system, we applied the net reclassification improvement (NRI) and the integrated discrimination improvement (IDI). A positive NRI and IDI indicated improved predictive ability, while negative values indicated a decrease.

Kaplan-Meier (K-M) analysis was used for time-dependent variables to calculate median survival times and plot OS and PFS curves, with group differences compared using the log-rank test. Hazard ratios (HRs) and 95% confidence intervals (CIs) were used to quantify relative risks. All the statistical analyses were performed using R 4.3.2 software. *P* values less than 0.05 were considered to indicate statistical significance.

## 3 Results

### 3.1 Patient characteristics

A total of 91 variables were included in this study, and the primary patient characteristics are summarized in **Table 1**. The majority of patients were male; aged between 50 and 69 years; had advanced T3-4 and N2-3 stages; had M1 status; had not undergone radical surgery or radiotherapy; and had received first-line treatment. Tumor characteristics predominantly included Her-2-negative status, poor differentiation, adenocarcinoma type, and tumors located in the upper stomach. The majority of treatment drugs used were sintilimab and camrelizumab, with combination therapy mainly involving immunotherapy combined with chemotherapy. There were no significant differences in any of the indices between the training and validation cohorts. **Supplementary Figure 1** presents the correlation heatmap of clinicopathological features and peripheral blood indices before and after immunotherapy for the 617 patients with advanced gastric/gastroesophageal junction cancer. Notably, a strong positive correlation was observed between pre-CA199 and post-CA199, as well as between pre-SIRI and pre-NLR.

**Table 1:** Clinicopathological characteristics of the patients.

Characteristic	Training cohort [cases (%)] (n = 433)	Validation cohort [cases (%)] (n = 184)	Total population [cases (%)] (n = 617)	*Р* value
Gender				0.272
Male	308 (71.1%)	122 (66.3%)	430 (69.7%)	
Female	125 (28.9%)	62 (33.7%)	187 (30.3%)	
Age (years)				0.258
<50	63 (14.5%)	23 (12.5%)	86 (13.9%)	
50-59	133 (30.7%)	71 (38.6%)	204 (33.1%)	
60-69	141 (32.6%)	50 (27.2%)	191 (31.0%)	
≥70	96 (22.2%)	40 (21.7%)	136 (22.0%)	
Smoking history				0.265
No	277 (64%)	127 (69%)	404 (65.5%)	
Yes	156 (36%)	57 (31%)	213 (34.5%)	
Alcohol history				1.000
No	330 (76.2%)	140 (76.1%)	470 (76.2%)	
Yes	103 (23.8%)	44 (23.9%)	147 (23.8%)	
Agent				0.901
Sintilimab	221 (51%)	88 (47.8%)	309 (50.1%)	
Camrelizumab	127 (29.3%)	57 (31%)	184 (29.8%)	
Tislelizumab	29 (6.7%)	15 (8.2%)	44 (7.1%)	
Toripalimab	15 (3.5%)	8 (4.3%)	23 (3.7%)	
Penpulimab	17 (3.9%)	5 (2.7%)	22 (3.6%)	
Nivolumab	12 (2.8%)	7 (3.8%)	19 (3.1%)	
Pembrolizumab	12 (2.8%)	4 (2.2%)	16 (2.6%)	
Combination				0.287
Chemotherapy	307 (70.9%)	133 (72.3%)	440 (71.3%)	
Targeted therapy	30 (6.9%)	18 (9.8%)	48 (7.8%)	
Chemotherapy + Targeted therapy	96 (22.2%)	33 (17.9%)	129 (20.9%)	
Treatment line				0.842
First line	313 (72.3%)	135 (73.4%)	448 (72.6%)	
Second line	95 (21.9%)	37 (20.1%)	132 (21.4%)	
Third or later	25 (5.8%)	12 (6.5%)	37 (6.0%)	
Radical surgery				0.776
No	338 (78.1%)	141 (76.6%)	479 (77.6%)	
Yes	95 (21.9%)	43 (23.4%)	138 (22.4%)	
Radiotherapy				0.051
No	425 (98.2%)	174 (94.6%)	599 (97.1%)	
Yes	8 (1.8%)	10 (5.4%)	18 (2.9%)	
BMI				0.088
Underweight (<18.5)	45 (10.4%)	27 (14.7%)	72 (11.7%)	
Normal (18.5-23.9)	259 (59.8%)	90 (48.9%)	349 (56.6%)	
Overweight (24-27.9)	99 (22.9%)	51 (27.7%)	150 (24.3%)	
Obese (≥28)	30 (6.9%)	16 (8.7%)	46 (7.5%)	
PD-L1 CPS				0.760
CPS < 1	96 (22.2%)	44 (23.9%)	140 (22.7%)	
CPS ≥ 1	154 (35.6%)	68 (37%)	222 (36.0%)	
Unknown	183 (42.3%)	72 (39.1%)	255 (41.3%)	
Her-2				0.068
Negative	291 (67.2%)	138 (75%)	429 (69.5%)	
Positive	69 (15.9%)	17 (9.2%)	86 (13.9%)	
Unknown	73 (16.9%)	29 (15.8%)	102 (16.5%)	
ki-67				0.853
<70%	108 (24.9%)	42 (22.8%)	150 (24.3%)	
≥70%	166 (38.3%)	73 (39.7%)	239 (38.7%)	
Unknown	159 (36.7%)	69 (37.5%)	228 (37.0%)	
Pathological type				1.000
Adenocarcinoma	406 (93.8%)	172 (93.5%)	578 (93.7%)	
Others	27 (6.2%)	12 (6.5%)	39 (6.3%)	
Differentiation degree				0.394
Poorly	262 (60.5%)	101 (54.9%)	363 (58.8%)	
Moderately and well	58 (13.4%)	26 (14.1%)	84 (13.6%)	
Unknown	113 (26.1%)	57 (31%)	170 (27.6%)	
Lauren classification				0.909
Intestinal type	57 (13.2%)	24 (13%)	81 (13.1%)	
Diffuse type	56 (12.9%)	24 (13%)	80 (13.0%)	
Mixed type	51 (11.8%)	18 (9.8%)	69 (11.2%)	
Unknown	269 (62.1%)	118 (64.1%)	387 (62.7%)	
Primary tumor site				0.446
Upper	235 (54.3%)	91 (49.5%)	326 (52.8%)	
Middle	96 (22.2%)	48 (26.1%)	144 (23.3%)	
Lower	89 (20.6%)	42 (22.8%)	131 (21.2%)	
Other	13 (3%)	3 (1.6%)	16 (2.6%)	
T stage				0.550
T1-T2	27 (6.2%)	11 (6%)	38 (6.2%)	
T3	141 (32.6%)	50 (27.2%)	191 (31.0%)	
T4	190 (43.9%)	91 (49.5%)	281 (45.5%)	
TX	75 (17.3%)	32 (17.4%)	107 (17.3%)	
N stage				0.219
N0	106 (24.5%)	52 (28.3%)	158 (25.6%)	
N1	34 (7.9%)	20 (10.9%)	54 (8.8%)	
N2	143 (33%)	47 (25.5%)	190 (30.8%)	
N3	150 (34.6%)	65 (35.3%)	215 (34.8%)	
M stage				0.816
M0	45 (10.4%)	21 (11.4%)	66 (10.7%)	
M1	388 (89.6%)	163 (88.6%)	551 (89.3%)	
Liver metastasis				0.171
No	281 (64.9%)	108 (58.7%)	389 (63.0%)	
Yes	152 (35.1%)	76 (41.3%)	228 (37.0%)	
Bone metastasis				0.416
No	405 (93.5%)	168 (91.3%)	573 (92.9%)	
Yes	28 (6.5%)	16 (8.7%)	44 (7.1%)	
Lymph node metastasis				0.475
No	66 (15.2%)	33 (17.9%)	99 (16.0%)	
Yes	367 (84.8%)	151 (82.1%)	518 (84.0%)	
Lung metastasis				0.851
No	394 (91%)	169 (91.8%)	563 (91.2%)	
Yes	39 (9%)	15 (8.2%)	54 (8.8%)	
Peritoneal metastasis				0.601
No	344 (79.4%)	142 (77.2%)	486 (78.8%)	
Yes	89 (20.6%)	42 (22.8%)	131 (21.2%)	
Ascites				0.327
No	364 (84.1%)	148 (80.4%)	512 (83.0%)	
Yes	69 (15.9%)	36 (19.6%)	105 (17.0%)	
Other metastases				0.855
No	355 (82%)	149 (81%)	504 (81.7%)	
Yes	78 (18%)	35 (19%)	113 (18.3%)	
Number of metastatic sites				0.584
0-1	167 (38.6%)	66 (35.9%)	233 (37.8%)	
2	165 (38.1%)	68 (37%)	233 (37.8%)	
≥3	101 (23.3%)	50 (27.2%)	151 (24.5%)	

### 3.2 Survival outcomes and efficacy evaluation

**Figure 2:**
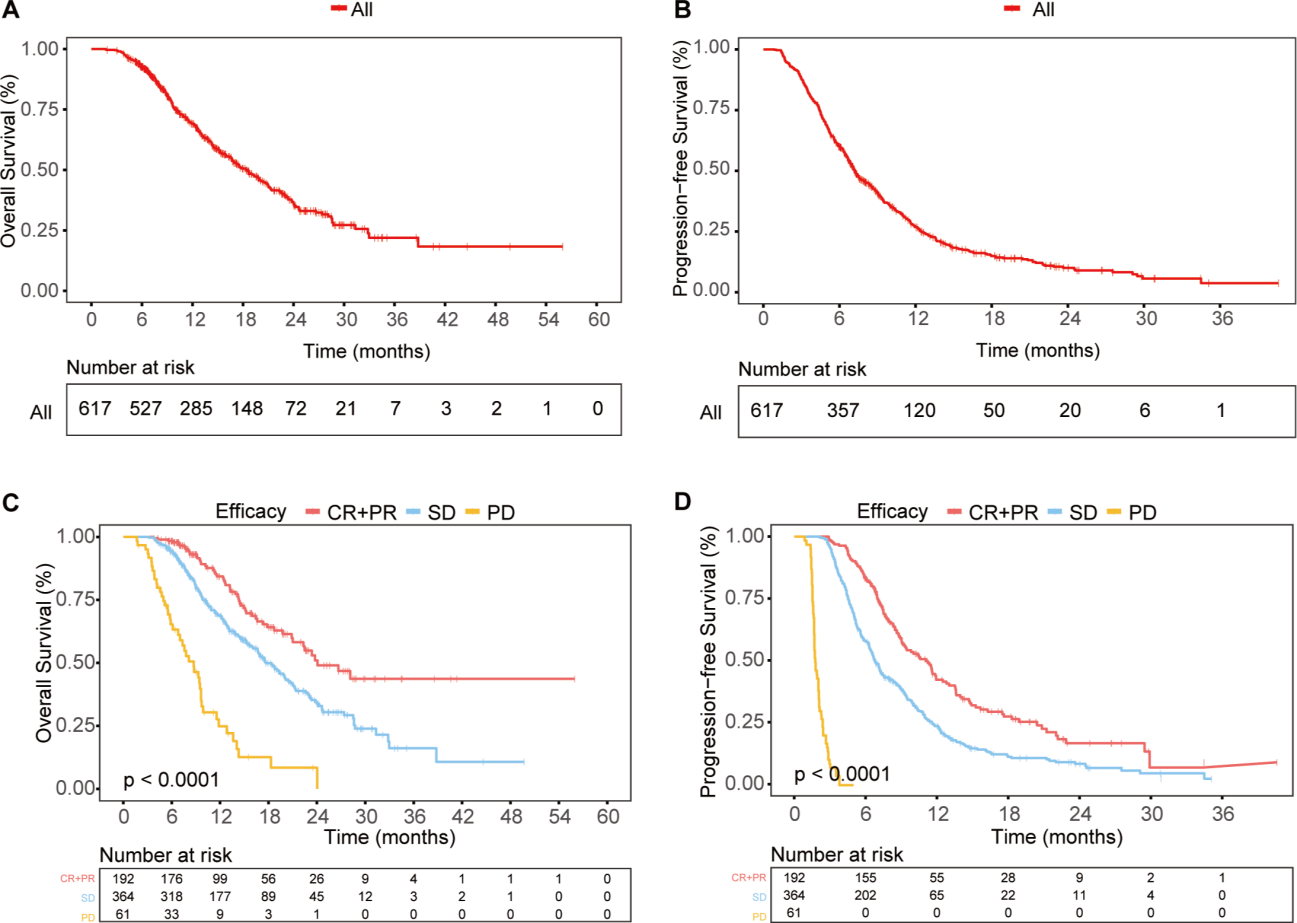
Survival outcomes in the total population **(A, B)** OS **(A)** and PFS **(B)** curves for the entire study population. **(C, D)** OS **(C)** and PFS **(D)** curves for different response groups: CR + PR, SD, and PD.

In the total study population, the median overall survival (mOS) was 18.37 months (95% CI: 16.47 - 20.27) (**Figure 2A**), and the median progression-free survival (mPFS) was 7.20 months (95% CI: 6.58 - 7.83) (**Figure 2B**). The ORR was 31.12%, and the DCR was 90.1%. Among the patients, 1 achieved CR, 191 achieved PR, 364 had SD, and 61 experienced progressive disease (PD). The clinical and peripheral blood characteristics of the patients in the CR+PR, SD, and PD groups are detailed in **Supplementary Table 1**. Significant differences were observed among these groups in terms of combined treatment regimens, treatment lines, PD-L1 expression, Her-2 expression, TNM stage, metastasis status, tumor markers, nutritional indices, inflammation indices, and so on. Notably, a greater proportion of patients in the CR+PR group than in the SD and PD groups received first-line treatment.

Furthermore, we investigated the correlation between early treatment response and long-term survival. The CR+PR, SD, and PD groups showed significant differences in survival, with patients who achieved CR or PR having the best OS and PFS, while those with PD had the worst outcomes (**Figures 2C, D**). This indicated that patients with better early treatment responses were more likely to have improved long-term survival.

### 3.3 Subgroup analysis based on PD-L1 expression, Her-2 status, and treatment lines

**Figure 3:**
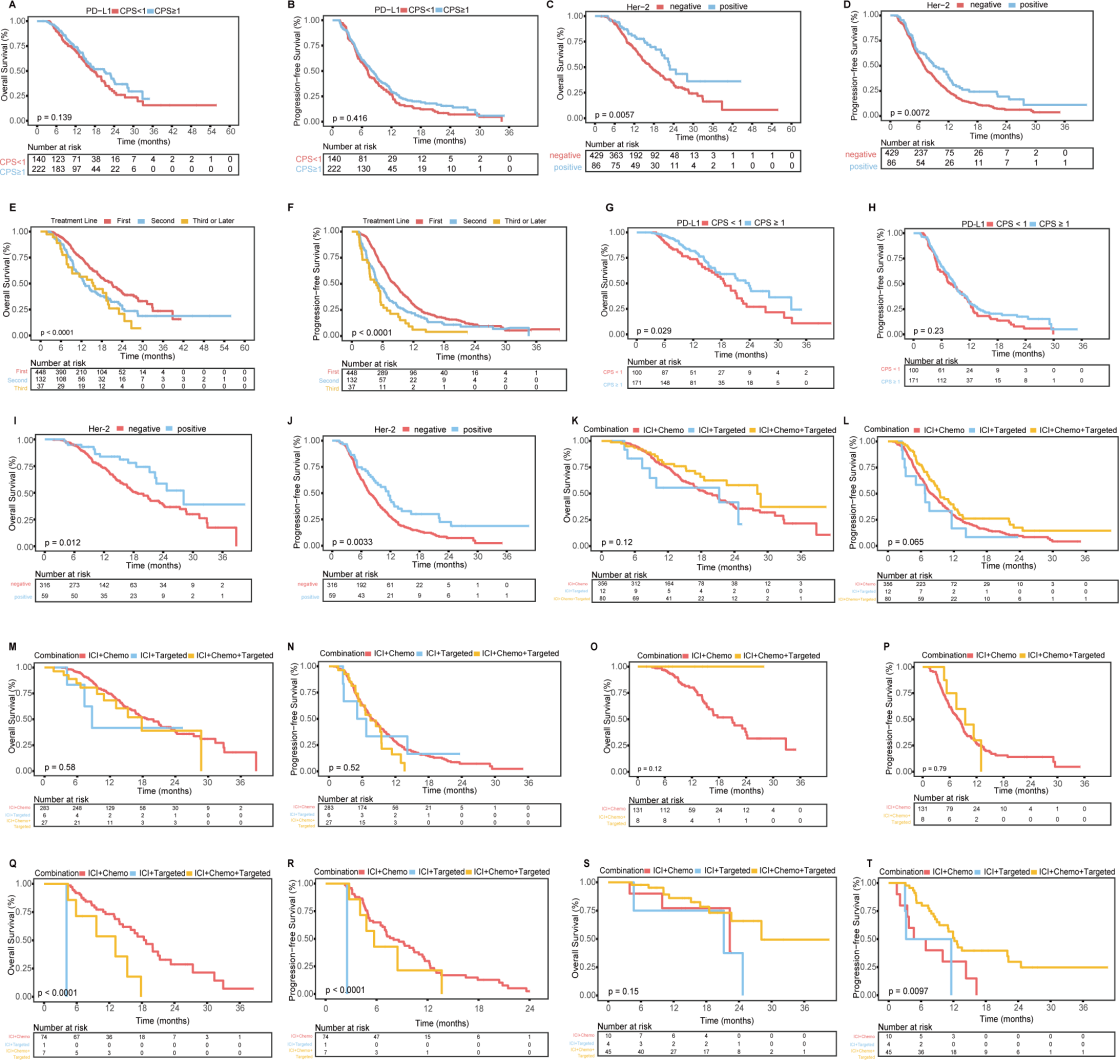
K-M curves related to PD-L1 expression, Her-2 expression, treatment lines, and treatment subgroups. **(A, B)** OS **(A)** and PFS **(B)** for patients with a PD-L1 CPS ≥1 vs. those with a CPS <1. **(C, D)** OS **(C)** and PFS **(D)** for Her-2-positive patients vs. Her-2-negative patients. **(E, F)** OS **(E)** and PFS **(F)** for patients receiving first-line vs. second-line vs. third-line or later treatments. **(G, H)** OS **(G)** and PFS **(H)** for first-line treatment subgroups of patients with a PD-L1 CPS ≥1 vs. those with a CPS <1. **(I, J)** OS **(I)** and PFS **(J)** for first-line treatment subgroups of Her-2-positive vs. Her-2-negative patients. **(K, L)** OS **(K)** and PFS **(L)** for first-line treatment subgroups receiving three different combined regimens. **(M, N)** OS **(M)** and PFS **(N)** for Her-2-negative patients receiving different first-line treatment regimens. **(O, P)** OS **(O)** and PFS **(P)** for Her-2-negative patients with a PD-L1 CPS ≥1 receiving different first-line treatment regimens. **(Q, R)** OS **(Q)** and PFS **(R)** for Her-2-negative patients with a PD-L1 CPS <1 receiving different first-line treatment regimens. **(S, T)** OS **(S)** and PFS **(T)** for Her-2-positive patients receiving different first-line treatment regimens.

Among the 362 patients with available PD-L1 expression data, the PD-L1 CPS ≥1 group exhibited a trend toward improved survival compared to the PD-L1 CPS <1 group, although no significant differences were observed in OS or PFS (**Supplementary Table 2**; **Figures 3A, B**).

Among the 515 patients with available Her-2 expression data, Her-2-positive patients had significantly better OS and PFS than Her-2-negative patients (**Supplementary Table 3**; **Figures 3C, D**).

When comparing first-line, second-line, and third-line or later treatments, first-line treatment was associated with significantly better OS and PFS than second-line and third-line treatments (**Supplementary Table 4**; **Figures 3E, F**).Given the widespread use of first-line anti-PD-1/PD-L1 treatment, we analyzed the first-line treatment group. Among the 448 patients, those in the PD-L1 CPS ≥1 group had significantly better OS than did those in the CPS <1 group, but there was no difference in PFS. Her-2-positive patients had better OS and PFS than Her-2-negative patients. No significant differences in OS or PFS were found among patients receiving different combinations of immunotherapy, chemotherapy, or targeted therapy. The detailed data are shown in **Figures 3G-L** and **Supplementary Table 5**.

For the 283 Her-2-negative patients who received first-line immunotherapy combined with chemotherapy, the mOS was 18.77 months (95% CI: 15.59–21.95) (**Figure 3M**), the mPFS was 7.77 months (95% CI: 6.79–8.75) (**Figure 3N**), the ORR was 34.63% (98/283), and the DCR was 95.76% (271/283). Further subgroup analysis based on the PD-L1 CPS is detailed in **Supplementary Table 6** (**Figures 3O-R**). Among the 45 Her-2-positive patients who received first-line immunotherapy combined with chemotherapy and targeted therapy, the mOS was 28.13 months (95% CI: 22.60-NA) (**Figure 3S**), the mPFS was 12.17 months (95% CI: 10.25-14.10) (**Figure 3T**), the ORR was 62.22% (28/45), and the DCR was 100% (45/45).

### 3.4 OS nomogram construction and validation

**Figure 4:**
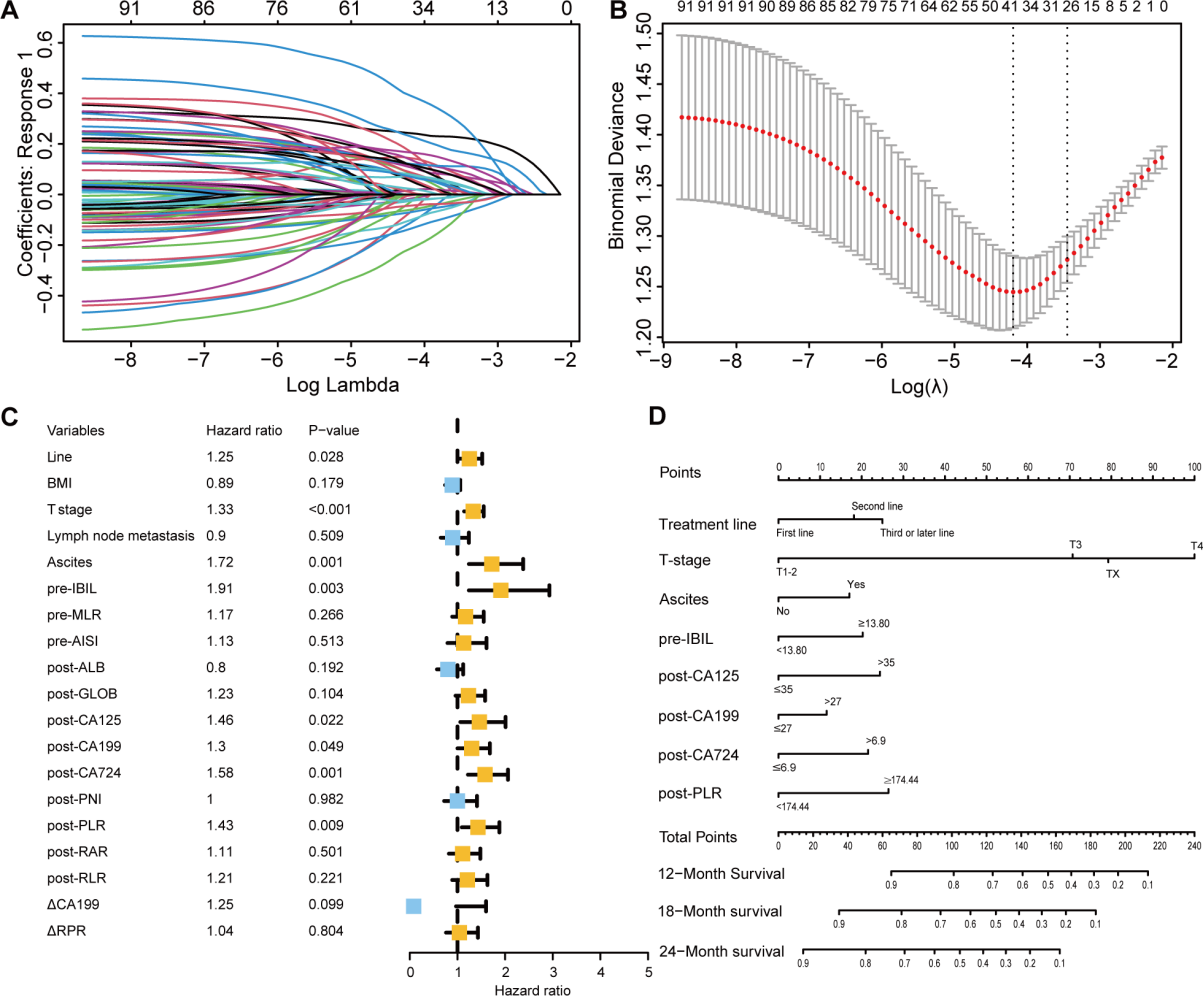
Selection of independent factors for OS and construction of the nomogram model **(A)** Overview of LASSO coefficients; **(B)** Selection of optimal parameters in the LASSO regression model; **(C)** Forest plot of multivariate Cox regression analysis results for OS; **(D)** Nomogram for predicting 12-month, 18-month, and 24-month OS rates.

Through LASSO-Cox regression analysis (**Figures 4A, B**), 8 independent factors associated with OS in patients receiving immunotherapy for advanced gastric cancer were identified, including treatment line, T stage, ascites, pretreatment indirect bilirubin (pre-IBIL), post-CA125, post-CA199, post-CA724, and post-PLR (**Table 2**). The multivariate Cox regression analysis results are presented in a forest plot (**Figure 4C**). Based on these eight factors, a nomogram was constructed to evaluate the 12-month, 18-month, and 24-month OS rates (**Figure 4D**). Each predictor has a corresponding risk score, and the total score estimates the patient’s survival probability. T stage was the primary factor affecting OS.

**Table 2:** Univariate and multivariate Cox regression analyses for OS.

Variable	Univariate Analysis			Multivariate Analysis		
	HR	95% CI	*P* value	HR	95% CI	*P* value
Treatment line	1.45	(1.22 - 1.72)	<0.001	1.25	(1.02 - 1.52)	0.028
BMI	0.82	(0.69 - 0.98)	0.026	0.89	(0.74 - 1.06)	0.179
Differentiation degree	0.88	(0.77 - 1.02)	0.091			
Lauren classification	0.92	(0.83 - 1.02)	0.099			
T stage	1.43	(1.24 - 1.63)	<0.001	1.33	(1.14 - 1.55)	<0.001
Lymph node metastasis	0.65	(0.48 - 0.87)	0.004	0.90	(0.65 - 1.24)	0.509
Ascites	2.16	(1.62 - 2.89)	<0.001	1.72	(1.24 - 2.38)	0.001
Other metastases	0.98	(0.71 - 1.35)	0.895			
pre-GLOB	1.34	(0.95 - 1.90)	0.095			
pre-IBIL	1.91	(1.29 - 2.85)	0.001	1.91	(1.24 - 2.93)	0.003
pre-MLR	1.45	(1.14 - 1.84)	0.002	1.17	(0.89 - 1.55)	0.266
pre-AISI	1.59	(1.19 - 2.15)	0.002	1.13	(0.79 - 1.61)	0.513
pre-RAR	1.10	(0.86 - 1.39)	0.453			
post-ALB	0.74	(0.58 - 0.94)	0.014	0.80	(0.57 - 1.12)	0.192
post-GLOB	1.29	(1.02 - 1.64)	0.035	1.23	(0.96 - 1.58)	0.104
post-DBIL	1.23	(0.91 - 1.65)	0.178			
post-CA125	2.24	(1.70 - 2.94)	<0.001	1.46	(1.06 - 2.01)	0.022
post-CA199	1.79	(1.41 - 2.28)	<0.001	1.30	(1.00 - 1.68)	0.049
post-CA724	1.85	(1.45 - 2.36)	<0.001	1.58	(1.22 - 2.06)	0.001
post-PNI	0.73	(0.57 - 0.92)	0.009	1.00	(0.72 - 1.41)	0.982
post-PLR	1.85	(1.46 - 2.35)	<0.001	1.43	(1.09 - 1.88)	0.009
post-RAR	1.30	(1.03 - 1.66)	0.030	1.11	(0.82 - 1.48)	0.501
post-RLR	1.35	(1.05 - 1.73)	0.018	1.21	(0.89 - 1.63)	0.221
△CA199	1.44	(1.13 - 1.82)	0.003	1.25	(0.97 - 1.60)	0.080
△NMR	1.25	(0.97 - 1.60)	0.082			
△RPR	0.75	(0.57 - 0.97)	0.031	1.04	(0.76 - 1.43)	0.804

To validate the model’s predictive accuracy, ROC curves, calibration curves, and the C-index were used. ROC curves showed AUCs for 12-month, 18-month, and 24-month OS rates of 0.759, 0.752, and 0.750, respectively, in the training cohort (**Supplementary Figure 2A**) and 0.749, 0.703, and 0.795, respectively, in the validation cohort (**Supplementary Figure 2B**), indicating excellent discriminative ability. Calibration curves confirmed that the predicted OS rates at 12, 18, and 24 months were consistent with the actual outcomes in both cohorts (**Supplementary Figures 2C-H**). The C-indices for the training and validation cohorts were 0.728 and 0.742, respectively, suggesting good model accuracy and precision. When the model was compared with the AJCC tumor staging system, DCA showed greater net benefit for the nomogram in both cohorts (**Supplementary Figure 3**). The C-index, NRI, and IDI results indicated a statistically superior ability to predict OS compared to that of the AJCC staging system (**Supplementary Table 7**).

**Figure 5:**
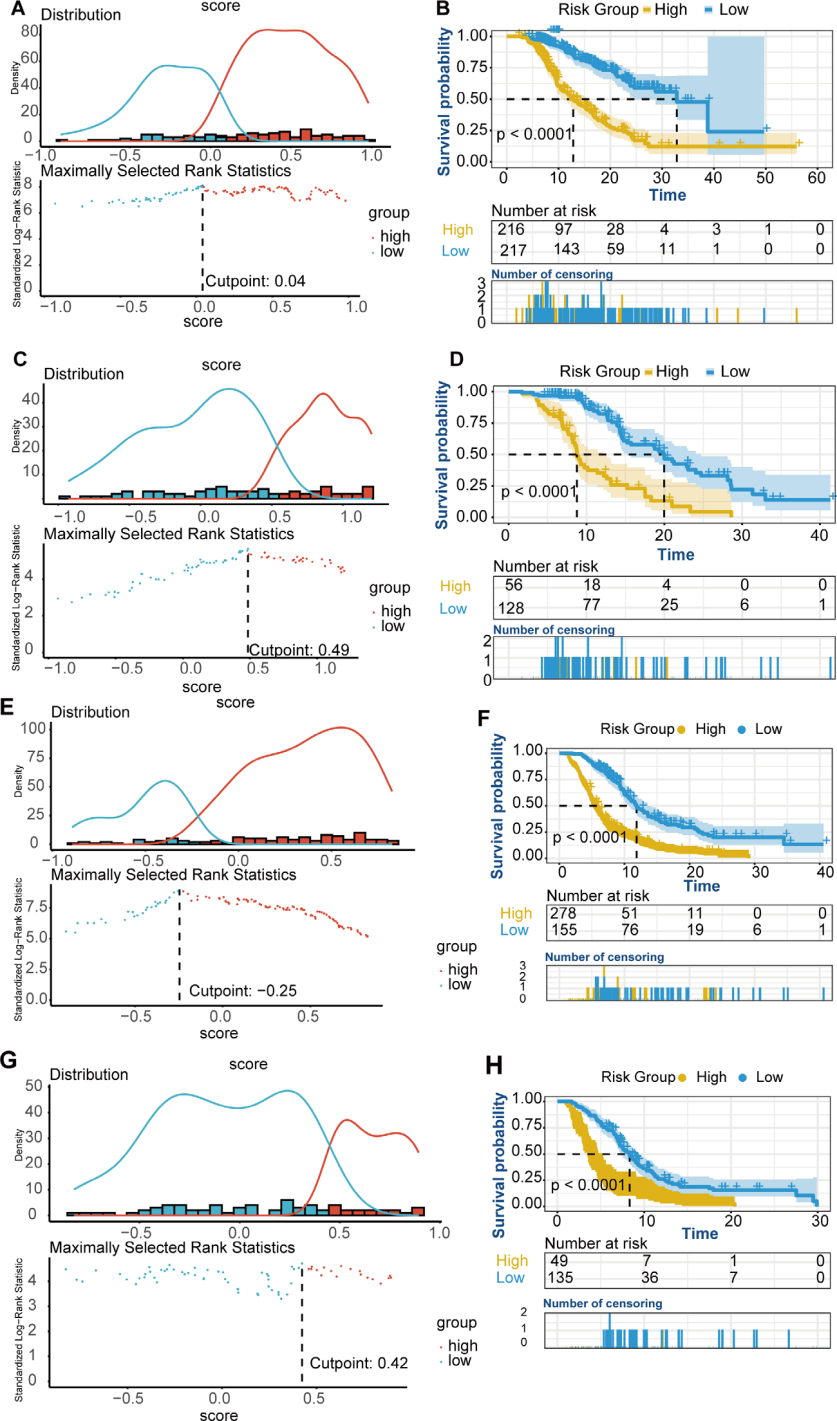
K-M curves for OS and PFS risk groups. **(A-D)** OS K-M curves for risk groups. **(A)** Risk stratification based on the OS nomogram score in the training cohort. **(B)** OS survival curves for high-risk and low-risk groups in the training cohort. **(C)** Risk stratification based on the OS nomogram score in the validation cohort. **(D)** OS survival curves for high-risk and low-risk groups in the validation cohort. **(E-H)** PFS K-M curves for risk groups. **(E)** Risk stratification based on the PFS nomogram score in the training cohort. **(F)** PFS survival curves for high-risk and low-risk groups in the training cohort. **(G)** Risk stratification based on the PFS nomogram score in the validation cohort. **(H)** PFS survival curves for high-risk and low-risk groups in the validation cohort.

Based on the nomogram scores, the population was stratified into high-risk and low-risk groups. K-M curves for OS revealed significantly better survival in the low-risk group in both cohorts, further confirming the effectiveness of the OS predictive nomogram (**Figures 5A-D**).

### 3.5 PFS nomogram construction and validation

**Figure 6:**
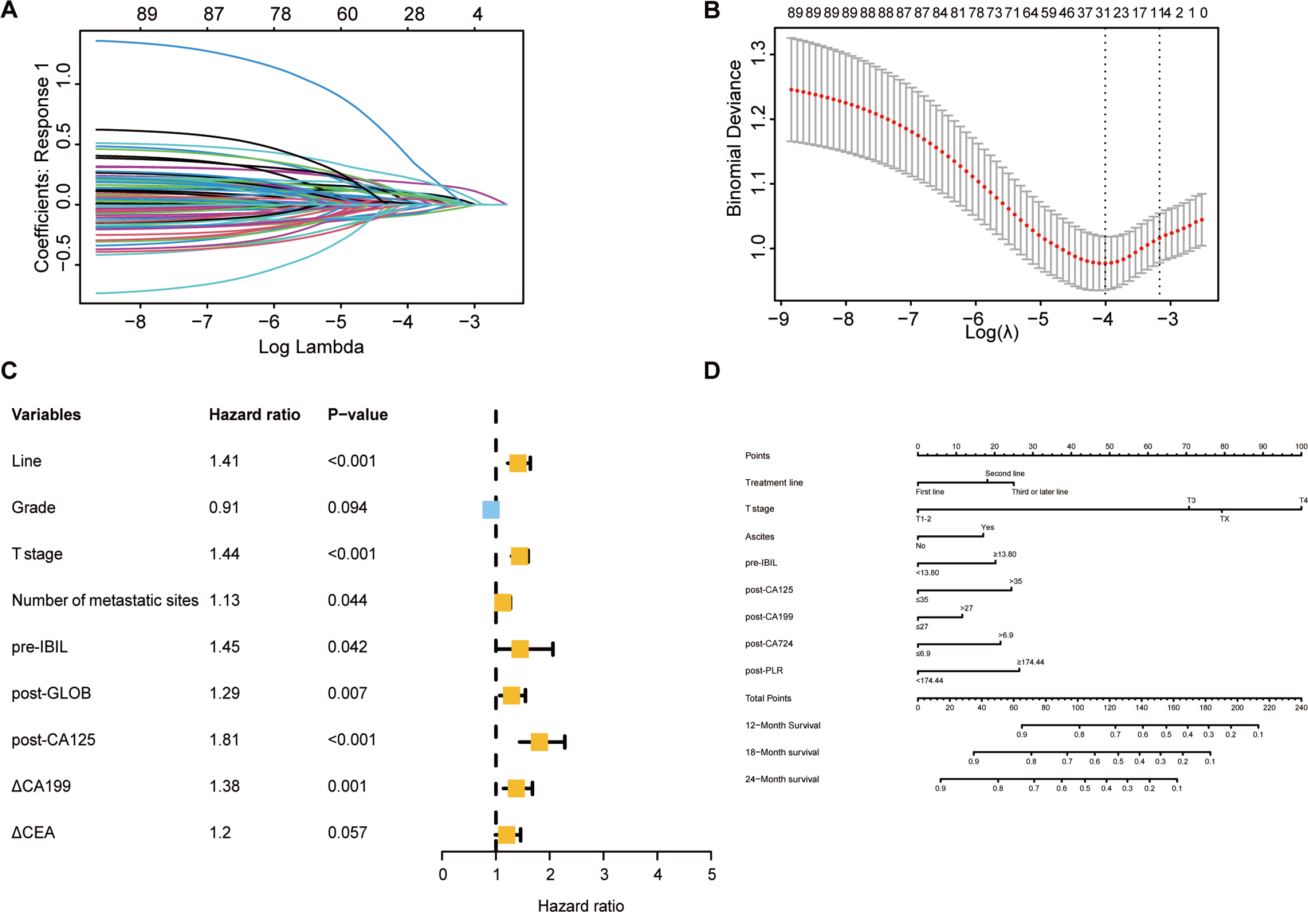
Selection of independent factors for PFS and construction of the nomogram model **(A)** Overview of LASSO coefficients; **(B)** Selection of the optimal parameters in the LASSO regression model; **(C)** Forest plot of multivariate Cox regression analysis for PFS; **(D)** Nomogram for predicting 6-month, 12-month, and 18-month PFS rates.

Using LASSO-Cox regression analysis (**Figures 6A, B**), seven independent factors associated with PFS in patients receiving immunotherapy for advanced gastric cancer, namely, treatment line, T stage, number of metastatic sites, pre-IBIL, post-GLOB, post-CA125, and △CA199, were identified (**Table 3**). The multivariate Cox regression analysis results are presented in a forest plot (**Figure 6C**). Based on these seven factors, a nomogram was constructed to evaluate the 6-month, 12-month, and 18-month PFS rates (**Figure 6D**). T stage was the primary factor affecting PFS, followed by treatment line.

**Table 3:** Univariate and multivariate Cox regression analyses for PFS.

Variable	Univariate Analysis			Multivariate Analysis		
	HR	95% CI	*P* value	HR	95% CI	*P* value
Treatment line	1.53	(1.32 - 1.76)	<0.001	1.41	(1.22 - 1.64)	<0.001
Differentiation degree	0.88	(0.79 - 0.97)	0.015	0.91	(0.82 - 1.02)	0.094
T stage	1.47	(1.33 - 1.63)	<0.001	1.44	(1.29 - 1.60)	<0.001
Number of metastatic sites	1.19	(1.06 - 1.33)	0.004	1.13	(1.00 - 1.27)	0.044
pre-IBIL	1.89	(1.34 - 2.66)	<0.001	1.45	(1.01 - 2.06)	0.042
post-GLOB	1.34	(1.12 - 1.60)	0.001	1.29	(1.07 - 1.55)	0.007
post-CA125	1.83	(1.47 - 2.28)	<0.001	1.81	(1.44 - 2.28)	<0.001
△CA199	1.48	(1.24 - 1.77)	<0.001	1.38	(1.14 - 1.68)	0.001
△CEA	1.35	(1.13 - 1.62)	<0.001	1.20	(0.99 - 1.46)	0.057

ROC curves and calibration curves were used to evaluate the model’s predictive ability. The ROC curves showed AUCs for 6-month, 12-month, and 18-month PFS rates of 0.764, 0.705, and 0.730, respectively, in the training cohort (**Supplementary Figure 4A**) and 0.730, 0.689, and 0.708, respectively, in the validation cohort (**Supplementary Figure 4B**). Calibration curves indicated that the predicted PFS rates at 6, 12, and 18 months were consistent with the actual outcomes in both cohorts (**Supplementary Figures 4C-H**). The DCA showed greater net benefit for the nomogram than for the AJCC staging system in both cohorts (**Supplementary Figure 5**). The C-index, NRI, and IDI results indicated that the nomogram had significantly superior clinical utility and effectiveness compared to the AJCC staging system (**Supplementary Table 8**).

Based on the nomogram scores, the population was stratified into high-risk and low-risk groups, with K-M curves for PFS showing significantly better survival in the low-risk group in both cohorts (**Figures 5E-H**).

### 3.6 ORR predictive model construction and evaluation

**Figure 7:**
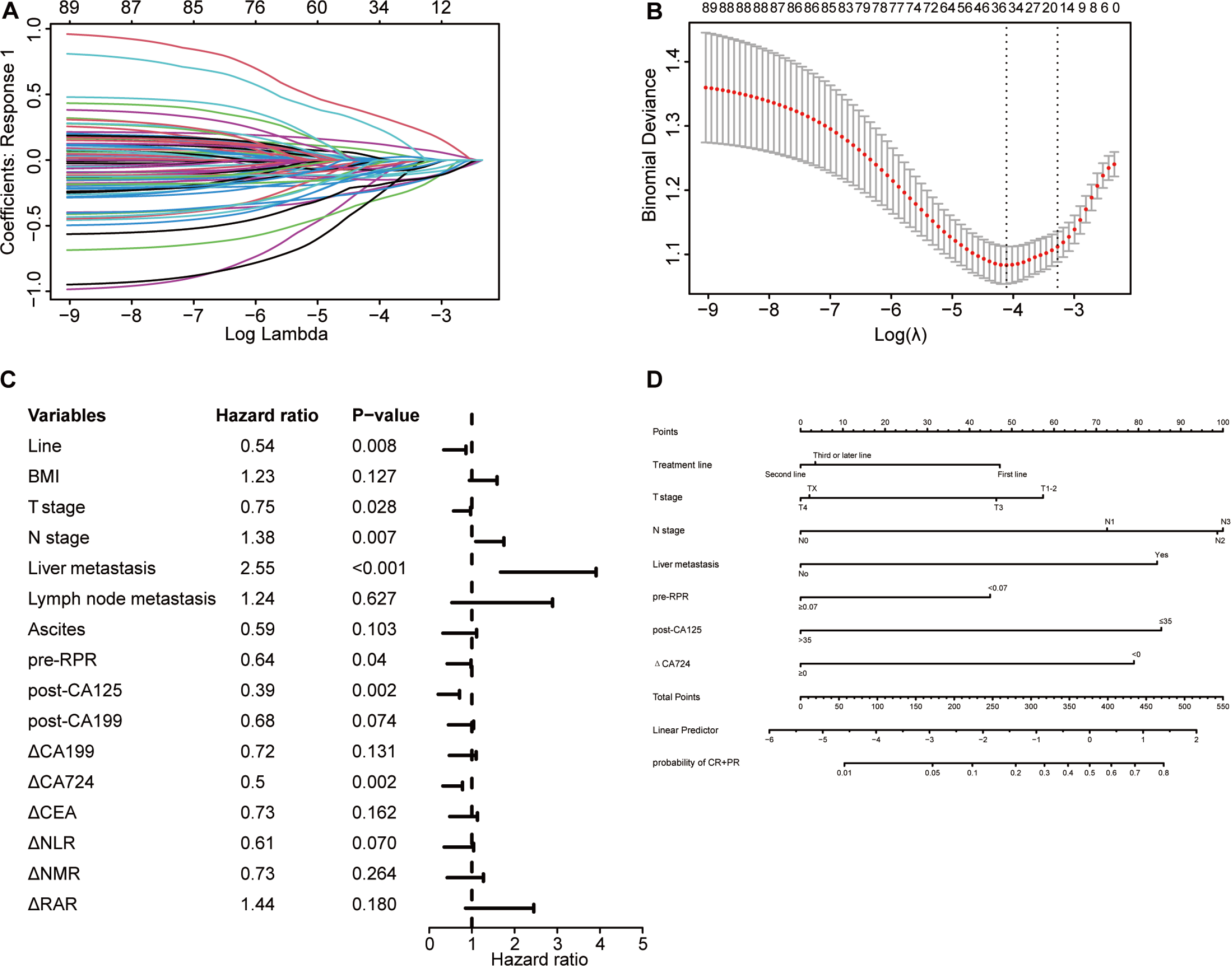
Selection of independent factors for ORR and construction of the nomogram model **(A)** Overview of LASSO coefficients; **(B)** Selection of optimal parameters in the LASSO regression model; **(C)** Forest plot of multivariate logistic regression analysis results for ORR; **(D)** Nomogram model predicting the probability of achieving CR or PR.

Using LASSO logistic regression analysis (**Figures 7A, B**), 7 independent factors associated with the ORR in patients receiving immunotherapy for advanced gastric cancer were identified, including treatment line, T stage, N stage, liver metastasis, pre-RPR, post-CA125, and △CA724 (**Table 4**). The multivariate logistic regression analysis results are presented in a forest plot (**Figure 7C**). Based on these seven predictors, a nomogram was constructed to evaluate the probability of achieving CR or PR (**Figure 7D**). N stage, post-CA125, liver metastasis, and △CA724 were the primary factors affecting the ORR. Each variable in the nomogram has a corresponding score, and the total score, calculated by summing all predictor scores, indicates a greater probability of achieving CR or PR with a higher total score. Earlier treatment, earlier T stage, later N stage, the presence of liver metastasis, lower pre-RPR, lower post-CA125 and decreased CA724 were associated with a greater probability of achieving CR or PR.

**Table 4:** Univariate and multivariate analysis of ORR.

Variable	Univariate Analysis			Multivariate Analysis		
	HR	95% CI	*P* value	HR	95% CI	*P* value
Treatment line	0.40	(0.27 - 0.59)	<0.001	0.54	(0.34 - 0.86)	0.008
BMI	1.44	(1.15 - 1.80)	0.002	1.23	(0.94 - 1.59)	0.127
T stage	0.57	(0.46 - 0.71)	<0.001	0.75	(0.57 - 0.97)	0.028
N stage	1.46	(1.25 - 1.71)	<0.001	1.38	(1.09 - 1.75)	0.007
Liver metastasis	2.15	(1.52 - 3.05)	<0.001	2.55	(1.67 - 3.91)	<0.001
Lymph node metastasis	3.86	(2.06 - 7.25)	<0.001	1.24	(0.53 - 2.89)	0.627
Lung metastasis	1.73	(0.98 - 3.05)	0.059			
Ascites	0.40	(0.23 - 0.69)	<0.001	0.59	(0.32 - 1.11)	0.103
pre-RPR	0.42	(0.30 - 0.59)	<0.001	0.64	(0.42 - 0.98)	0.040
post-CA125	0.42	(0.25 - 0.70)	<0.001	0.39	(0.21 - 0.71)	0.002
post-CA199	0.64	(0.44 - 0.91)	0.014	0.68	(0.45 - 1.04)	0.074
△CA199	0.54	(0.38 - 0.78)	<0.001	0.72	(0.47 - 1.10)	0.131
△CA724	0.45	(0.31 - 0.66)	<0.001	0.50	(0.32 - 0.78)	0.002
△CEA	0.48	(0.34 - 0.68)	<0.001	0.73	(0.48 - 1.13)	0.162
△NLR	0.37	(0.24 - 0.57)	<0.001	0.61	(0.35 - 1.04)	0.070
△NMR	0.43	(0.28 - 0.65)	<0.001	0.73	(0.42 - 1.27)	0.264
△RAR	2.04	(1.32 - 3.14)	0.001	1.44	(0.85 - 2.45)	0.180

**Figure 8:**
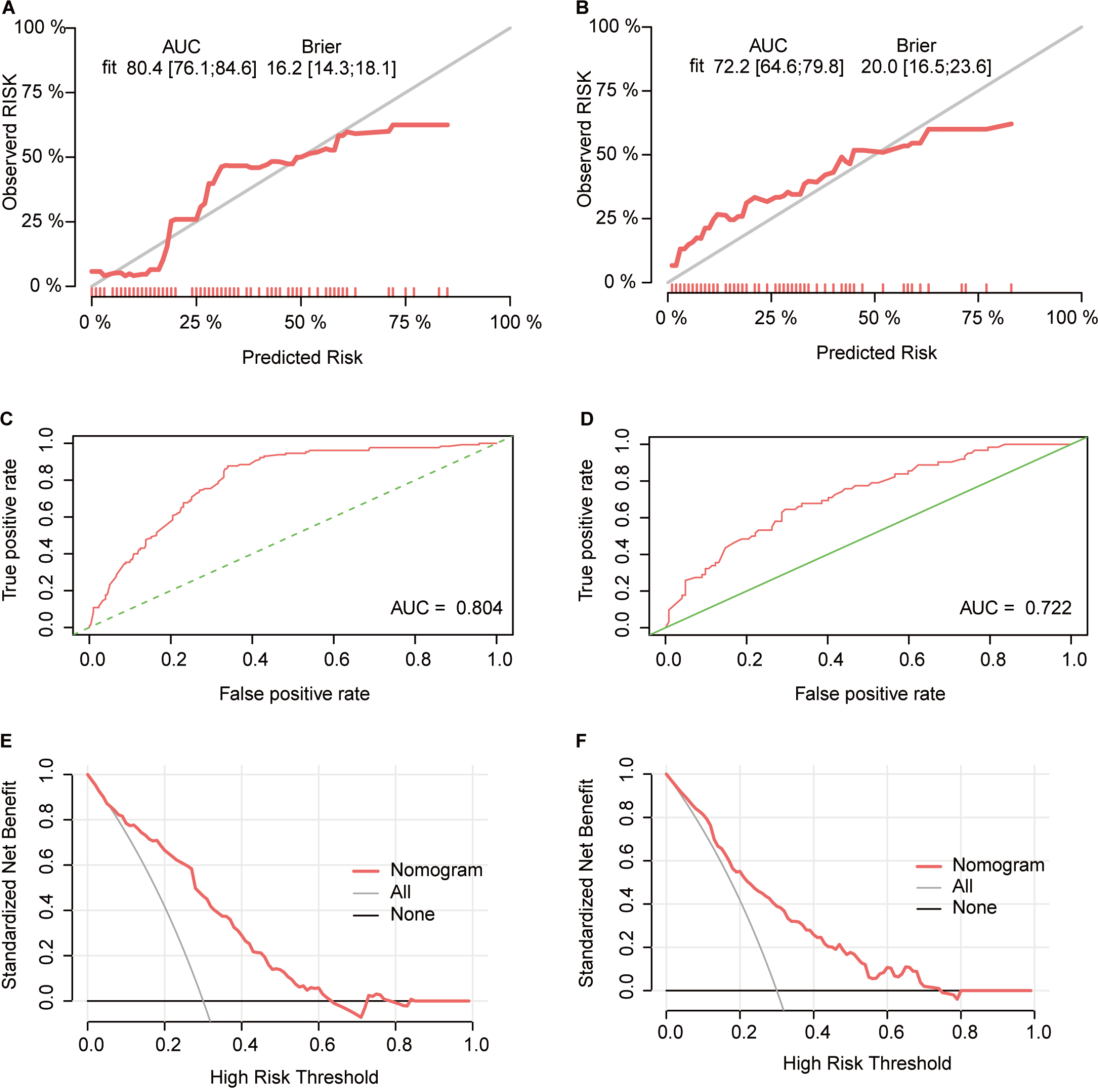
Validation of the ORR predictive nomogram model **(A, B)** Calibration curves for the CR+PR rate in the training **(A)** and validation **(B)** cohorts; **(C, D)** ROC curves of the treatment response nomogram in the training **(C)** and validation **(D)** cohorts; **(E, F)** DCA curve in the training **(E)** and validation **(F)** cohorts.

To better evaluate the nomogram’s predictive value, calibration curves, ROC curves, and decision curves were plotted. Calibration curves showed that the predicted probabilities were consistent with the actual outcomes in the training and validation cohorts (**Figures 8A, B**). The ROC curves showed AUCs of 0.804 in the training cohort and 0.722 in the validation cohort (**Figures 8C, D**), indicating excellent predictive accuracy. Decision curves indicated good clinical utility of the model (**Figures 8E, F**). These results confirmed that the nomogram is a simple yet effective model for predicting therapeutic response in advanced gastric cancer patients receiving immunotherapy.

According to the ORR predictive nomogram, a higher N stage and liver metastasis were paradoxically associated with a greater probability of achieving CR or PR. K-M analysis revealed no significant difference in OS among patients with different N stages (**Supplementary Figure 8E**), but a significant difference in PFS was observed (**Supplementary Figure 9E**). No significant difference in OS was found between patients with and without liver metastasis (**Supplementary Figure 8F**), but those without liver metastasis had significantly better PFS (**Supplementary Figure 9F**). Further exploration of potential causes revealed differences in clinicopathological characteristics between patients with different N stages (**Supplementary Table 9**) and between patients with and without liver metastasis (**Supplementary Table 10**). The greater proportion of patients receiving combined chemotherapy and targeted therapy due to higher PD-L1 CPS≥1 and Her-2 positivity rates, along with the greater proportion of first-line treatment in N2-3 stage patients, might explain the greater probability of achieving CR or PR in these patients. In addition, N0 patients had a significantly lower BMI than patients in other N stages, indicating poorer nutritional status, which may have affected their treatment outcomes. A greater proportion of patients with liver metastasis without peritoneal metastasis or ascites than without liver metastasis had liver metastasis, and their BMI was greater. Additionally, patients with liver metastasis had a greater prevalence of the intestinal type and a lower prevalence of the diffuse type, whereas those without liver metastasis had the opposite pattern. Previous studies have confirmed that the prognosis for diffuse-type gastric adenocarcinoma is generally worse than that for intestinal-type gastric adenocarcinoma (35).

### 3.7 Survival analysis

K-M analysis demonstrated survival differences for OS and PFS. OS predictors included treatment line (**Figures 3E, F**), T stage (**Supplementary Figure 6A**), ascites (**Supplementary Figure 6B**), pre-IBIL (**Supplementary Figure 6C**), post-CA125 (**Supplementary Figure 6D**), post-CA199 (**Supplementary Figure 6E**), post-CA724 (**Supplementary Figure 6F**), and post-PLR (**Supplementary Figure 7A**). Early treatment, early T stage, no ascites, and lower levels of pre-IBIL, post-CA125, post-CA199, post-CA724, and post-PLR were associated with longer OS. PFS predictors included treatment line (**Figure 3**), T stage (**Supplementary Figure 8A**), metastatic site (**Supplementary Figure 9B**), pre-IBIL (**Supplementary Figure 9A**), post-GLOB (**Supplementary Figure 9C**), post-CA125 (**Supplementary Figure 8D**), and △CA199 (**Supplementary Figure 9D**). Fewer metastatic sites, lower post-GLOB, and decreased △CA199 were associated with better PFS.

## 4 Discussion

Currently, for Her-2-negative, unresectable, advanced or recurrent gastric or gastroesophageal junction cancer patients, first-line treatment with a combination of anti-PD-1/PD-L1 therapy and chemotherapy is recommended. This recommendation is based on several large phase III clinical trials, including the ATTRACTION-4 study (36), CheckMate 649 study (37), KEYNOTE-859 study (38), ORIENT-16 study (39), and Rationale 305 study (68). In our study, for Her-2-negative patients receiving first-line immunotherapy combined with chemotherapy, the mOS and mPFS were 18.77 months and 7.77 months, respectively, with an ORR of 34.63% and a DCR of 95.76%. Although our study’s OS and PFS results were comparable to or even better than those of large phase III trials, the ORR was not as high. Since this study reflected real-world clinical practice, a high proportion of patients had distant metastases, relatively poor baseline conditions, and significant tumor burden, which may have contributed to the poor ORR observed in this study. Additionally, the superior OS results in this study may be partly explained by the fact that patients often adjust their treatment regimens and continue comprehensive therapy after the failure of first-line immunotherapy.

We also analyzed PD-L1 expression in Her-2-negative patients receiving first-line immunotherapy combined with chemotherapy. For patients with a PD-L1 CPS ≥1, the mOS was 20.87 months, the mPFS was 7.97 months, the ORR was 42.75%, and the DCR was 95.42%, outperforming the results from the CheckMate 649 and KEYNOTE-859 studies (37, 38). Currently, the role of PD-L1 expression in predicting the efficacy of immunotherapy is inconsistent. Although this study did not observe significant differences in OS or PFS between the PD-L1 CPS ≥1 and CPS <1 groups, the survival curves of the CPS ≥1 group showed a trend toward better outcomes than did those of the CPS <1 group. The CheckMate 649, KEYNOTE-859, ORIENT-16, and RATIONALE 305 studies demonstrated that nivolumab, pembrolizumab, sintilimab, and tislelizumab combined with chemotherapy provided survival benefits regardless of PD-L1 expression in the overall population. However, the ATTRACTION-4 study showed that patients with tumor cell PD-L1 expression ≥1% had shorter OS and PFS than did those with undefined or <1% PD-L1 expression. Therefore, it is still unclear whether the efficacy and survival advantage of gastric cancer immunotherapy increase with increasing PD-L1 expression levels, and the use of PD-L1 alone as a biomarker to predict immunotherapy efficacy is not accurate. A meta-analysis suggested that a PD-L1 CPS ≥1 was a critical threshold for survival benefit with immunotherapy alone, while immunotherapy combined with other therapies extended PFS and OS in all populations. In addition, the ORR was not affected by the PD-L1 CPS (40).

According to the analysis of Her-2 expression, Her-2-positive patients had significantly better OS and PFS than Her-2-negative patients, suggesting a potential benefit from combining immunotherapy with anti-Her-2 targeted therapy and chemotherapy. This hypothesis is supported by the KEYNOTE-811 study, which demonstrated that pembrolizumab combined with trastuzumab and chemotherapy significantly improved survival in advanced HER-2-positive gastric or gastroesophageal junction adenocarcinoma patients (41).

Additionally, we explored the relationship between recent treatment response and long-term survival. Patients who achieved CR or PR had significantly extended OS and PFS. The CheckMate 649 study explored the survival of patients with different response levels in the field of first-line immunotherapy for gastric cancer and revealed that Chinese patients (PD-L1 CPS ≥5) who achieved CR or PR at 18 weeks with nivolumab combined with chemotherapy had a 3-year OS rate of 37% and an mOS of 21.5 months (42). This indicated that achieving tumor shrinkage with immunotherapy likely led to longer survival. However, ORR and OS are not absolutely correlated. For example, several phase III studies in the field of gastric cancer immunotherapy have not achieved statistically significant OS benefits despite significant ORR benefits (41, 43). Additionally, the ability of different therapies to translate ORR benefits into long-term survival varies. For example, in the CheckMate 649 study, patients who achieved CR or PR in the chemotherapy group (PD-L1 CPS ≥5) had a 3-year OS rate of only 14% (44).

We observed that treatment line and T stage were independent predictors of OS, PFS, and ORR, which has been preliminarily confirmed in previous studies (45, 46). Since immunotherapy primarily enhances the antitumor immune response to kill tumor cells, theoretically, the earlier immunotherapy is applied, the better the effect. A meta-analysis of 25 clinical trials involving 20,013 patients with NSCLC also confirmed this hypothesis, showing that patients who received immunotherapy first and other treatments after failure had significantly longer OS than did those who received other treatments first and immunotherapy after failure, with a greater than 30% reduction in the risk of death (47). Our study also revealed that ascites and multiple organ metastases were associated with poor prognosis, consistent with previous studies (48). According to a Chinese subgroup analysis of the CheckMate 649 study, immunotherapy showed great therapeutic advantages for patients with peritoneal and liver metastases (42). Data from the PD-L1 CPS ≥5 subgroup showed that in the peritoneal metastasis group, nivolumab combined with chemotherapy achieved an mOS of 14.8 months, nearly three times that of the chemotherapy group; in the liver metastasis group, nivolumab combined with chemotherapy achieved an mOS of 14.3 months, nearly double that of the chemotherapy group.

In this study, several tumor markers exhibited strong predictive capabilities. Numerous studies have reported associations between baseline or dynamic serum tumor marker levels and immunotherapy efficacy (23–25, 49). Combining multiple tumor markers can increase the diagnostic sensitivity for gastric cancer and better predict its prognosis (50, 51). In a study of 146 patients with gastric cancer receiving chemotherapy or immunotherapy, CA724 was confirmed to be an independent prognostic factor for PFS and OS. The role of tumor markers in gastric cancer immunotherapy may be underreported, possibly because most studies have focused on baseline tumor marker data. In our study, meaningful data included tumor marker indices after two cycles of immunotherapy and changes before and after treatment. We found that if tumor marker levels decrease from baseline after immunotherapy, patients might achieve better treatment efficacy and survival, providing new insights for subsequent research.

Our study revealed that pre-IBIL was an independent predictor of OS and PFS. The baseline IBIL concentration has been confirmed to be an independent prognostic factor for OS in gastric cancer patients receiving ICIs or chemotherapy but has not been studied in a cohort of patients exclusively receiving immunotherapy for gastric cancer (46). This finding fills that gap. Additionally, low levels of ALB or high levels of GLOB in many types of cancer are often associated with high mortality and recurrence rates (52–55). High levels of globulin are caused by an increase in acute phase proteins and immunoglobulins and are believed to be associated with tumor proliferation, immune evasion, and distant metastasis (56). Some studies have shown that baseline GLOB is a predictor of tumor-specific survival in gastric cancer patients, but multivariate analysis did not reveal an association between globulin levels and prognosis (57). In our study, post-GLOB was an independent predictor of PFS in advanced gastric cancer patients receiving immunotherapy.

Several retrospective studies and meta-analyses have suggested that a low pretreatment PLR may be a potential favorable prognostic biomarker for the survival of patients with various cancers, including gastric cancer (49, 58–61). In patients with advanced and metastatic gastric cancer receiving immunotherapy, pretreatment PLR was significantly associated with PFS and OS (62, 63). This study revealed that the posttreatment PLR might be an independent predictor of OS, providing new ideas for future research. We speculate that a high PLR is associated with poor OS because platelet activation is present at all stages of tumor development, spread, and metastasis (64). When tumor cells enter the bloodstream, platelets aggregate on their surface, protecting tumor cells from attack by immune cells. Platelets also promote tumor metastasis and angiogenesis by releasing various growth factors, such as vascular endothelial growth factor-A, and can promote immune evasion and chemoresistance in tumor cells (17). On the other hand, an increase in lymphocytes is also associated with increased sensitivity to ICIs (65). Therefore, an elevated PLR indicates a cellular environment highly conducive to tumor growth and a poor response to immunotherapy. Notably, other inflammatory composite indices, such as the NLR, MLR, NMR, SII, NLPR, AISI, and SIRI, did not show potential for predicting treatment efficacy or survival in this study. Therefore, the practical application of inflammatory markers in the clinic should still be approached with caution.

PLT and RDW have been confirmed to be associated with the prognosis of cancer patients, but both indicators are easily affected by diseases other than tumors (66). In contrast, the RPR may be a more reliable indicator of treatment efficacy and patient prognosis and has been confirmed to reflect the severity of tumors (67). In this study, the pre-RPR was found to be an independent predictor of the ORR in patients receiving advanced gastric cancer immunotherapy, demonstrating the potential of the RPR, which is distinct from the findings of previous studies.

By collecting a large sample of real-world patient data, which includes comprehensive clinicopathological characteristics and peripheral blood indicators, our study has constructed a robust and practical model for predicting the efficacy and survival of gastric cancer patients receiving immunotherapy. We integrated both baseline and post-treatment peripheral blood data, assessing changes after two treatment cycles. This dynamic analysis provides valuable insights into the potential of blood-based biomarkers for guiding immunotherapy in gastric cancer patients. Moreover, while prior studies have predominantly focused on PFS and OS, our research uniquely addresses the ORR, offering the first nomogram prediction models related to ORR in this context. This novel aspect of our study fills a crucial gap in the current literature, further enhancing its clinical relevance.

This study also has several limitations. First, although this was a multicenter clinical study, the uneven geographic distribution of hospitals may limit the generalizability of the findings. Second, due to inconsistent routine examinations in different hospitals, the completeness of the data is limited, and there are patients with unknown PD-L1 CPS, Her-2, and Ki-67 status, which may cause statistical bias. Third, the selection of ICIs in this study was not uniform. Therefore, to obtain higher-level medical evidence, larger sample prospective studies are needed. Fourth, the follow-up time for patients in this study was relatively short, and the nomogram can predict OS rates up to 2 years. Longer follow-up periods are needed to analyze the 3-year and 5-year survival rates and long-term prognosis of patients.

## 5 Conclusions

This study highlights several important findings regarding the clinical outcomes of advanced gastric or gastroesophageal junction cancer patients treated with PD-1/PD-L1 inhibitors. Earlier treatment, lower T stage, absence of ascites, and lower levels of pre-IBIL, post-CA125, post-CA199, post-CA724, and post-PLR were associated with better OS. PFS was improved in patients with earlier treatment, lower T stage, fewer metastatic sites, and lower levels of pre-IBIL, post-GLOB, and post-CA125. Additionally, patients with earlier treatment, lower T and N stages, absence of liver metastases, and lower pre-RPR and post-CA125 levels were more likely to achieve a favorable objective response. Our validated nomogram model based on these indicators offers a practical tool for identifying patients most likely to benefit from immunotherapy, providing valuable clinical guidance for personalized treatment strategies.

## Data Availability

The original contributions presented in the study are included in the article/**Supplementary Material**. Further inquiries can be directed to the corresponding author.
