# Supplementary material for: Analysis of the treatment efficacy and prognostic factors of PD-1/PD-L1 inhibitors for advanced gastric or gastroesophageal junction cancer: a multicenter, retrospective clinical study
Source: Front Immunol. 2024 Oct 24;15:1468342. doi: 10.3389/fimmu.2024.1468342 (PMC11540680; doi:10.3389/fimmu.2024.1468342)
Supplement: Supplementary file 1 [file Presentation1.pdf]

1 Supplementary Figures

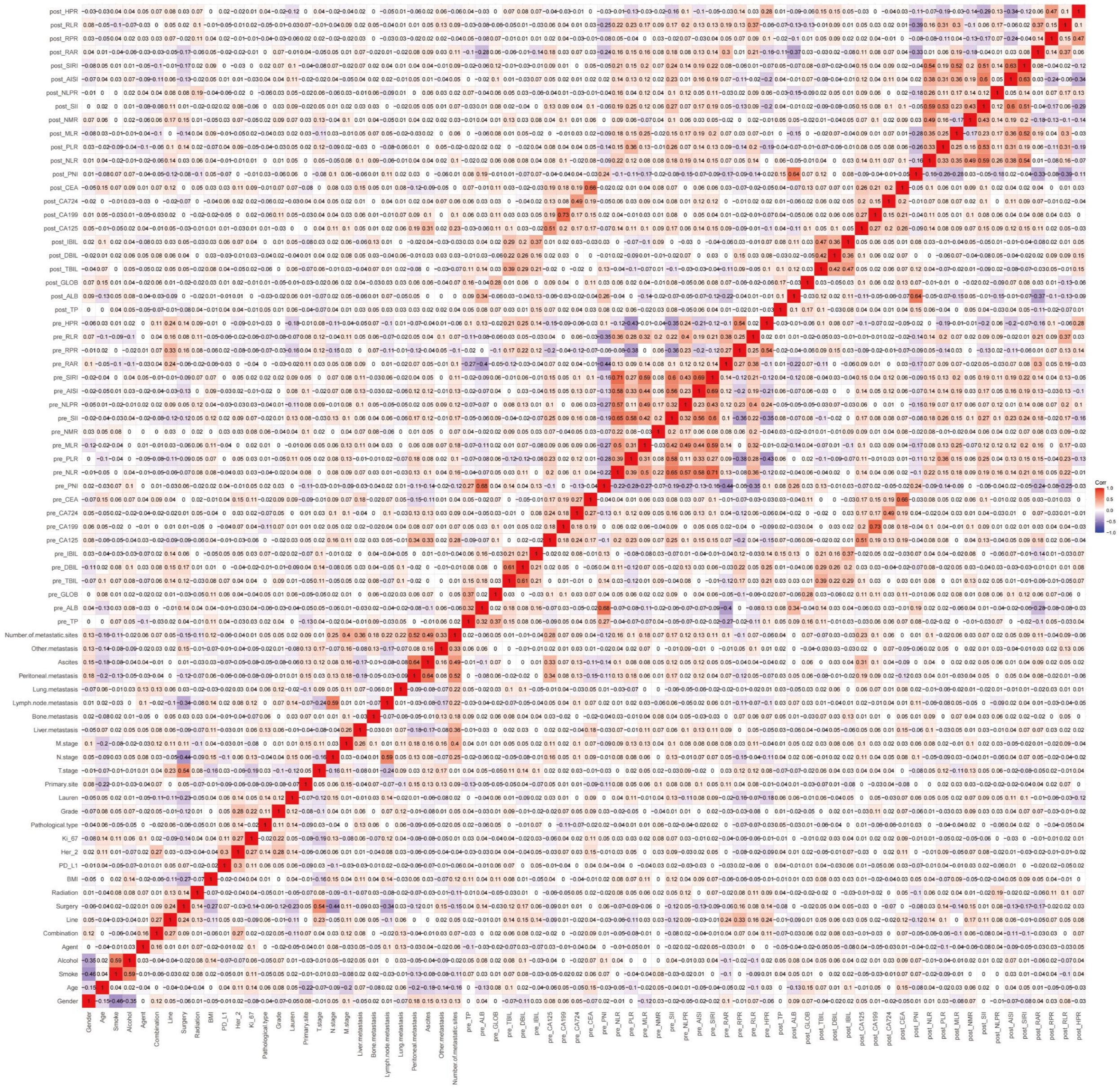

Supplementary Figure 1. Heatmap of correlations among all clinicopathological features and peripheral blood indices

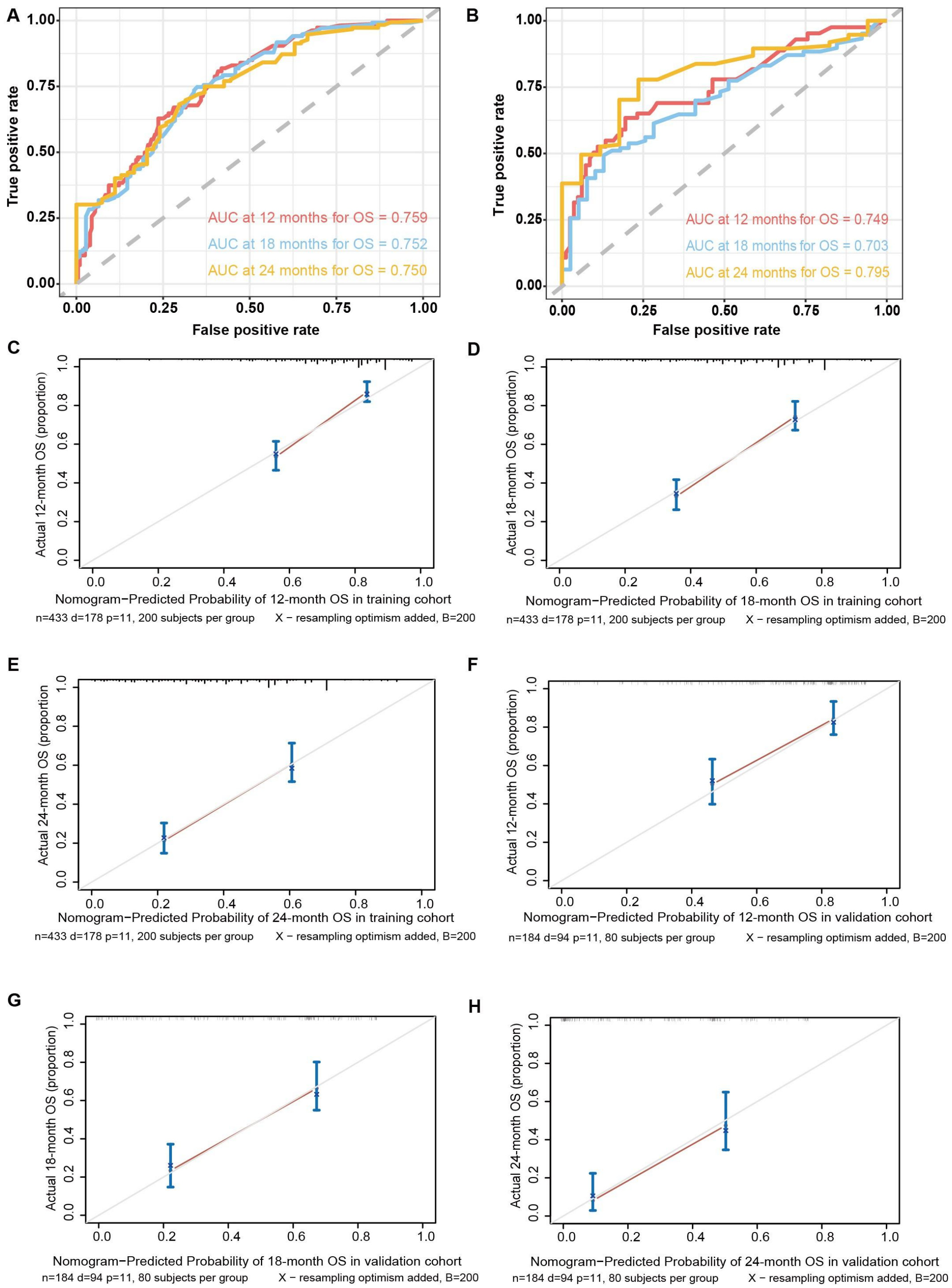

Supplementary Figure 2. ROC and calibration curves for the OS nomogram (A-B): ROC curves for the OS nomogram in the training **(A)** and validation **(B)** cohorts; **(C-H):** Calibration curves for the 12-month **(C, F)**, 18-month **(D, G)**, and 24-month **(E, H)** OS rates of the OS nomogram in the training cohort **(C-E)** and validation cohort **(F-H)**. The X-axis represents the predicted survival, and the Y-axis represents the actual survival. The bars indicate the 95% confidence intervals, and the dashed line indicates the reference line.

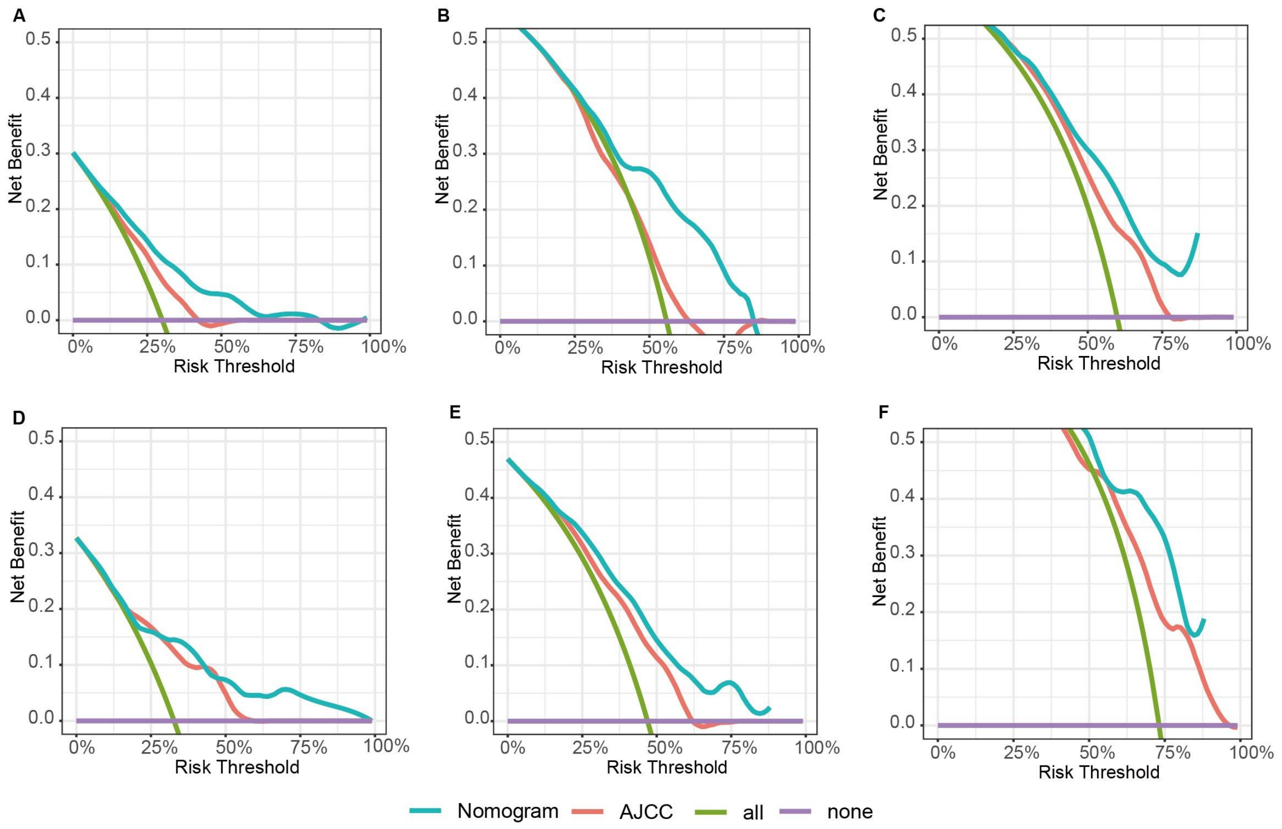

Supplementary Figure 3. DCA curves based on the nomogram model and AJCC tumor staging system (A-C): DCA curves for 12-month **(A)**, 18-month **(B)**, and 24-month **(C)** OS benefit in the training cohort; **(D-F):** DCA curves for 12-month **(D)**, 18-month **(E)**, and 24-month **(F)** OS benefit in the validation cohort.

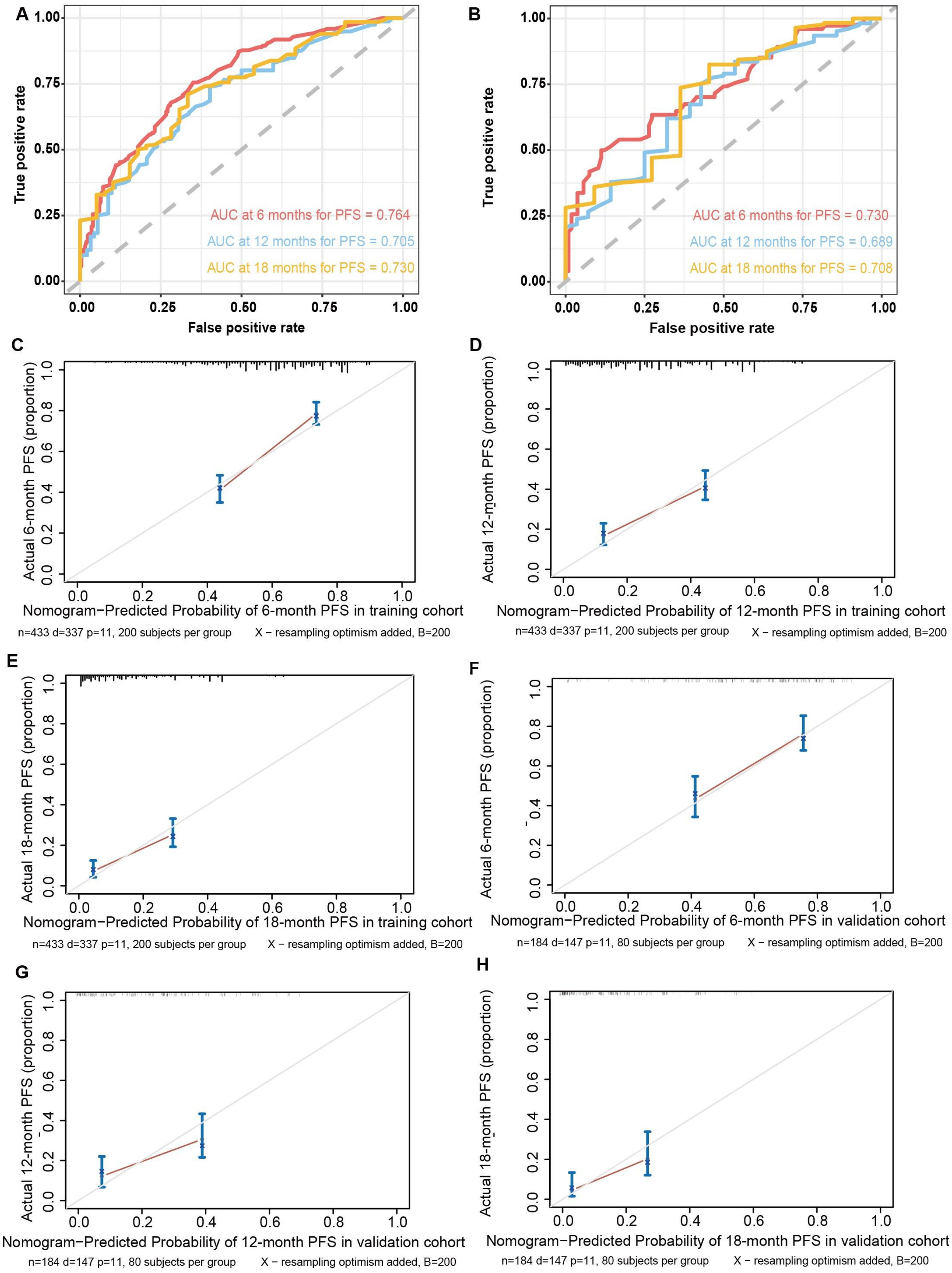
A
True positive rate
False positive rate
AUC at 6 months for PFS = 0.764
AUC at 12 months for PFS = 0.705
AUC at 18 months for PFS = 0.730
B
True positive rate
False positive rate
AUC at 6 months for PFS = 0.730
AUC at 12 months for PFS = 0.689
AUC at 18 months for PFS = 0.708
C
Actual 6-month PFS (proportion)
Nomogram−Predicted Probability of 6-month PFS in training cohort
n=433 d=337 p=11, 200 subjects per group
X − resampling optimism added, B=200
D
Actual 12-month PFS (proportion)
Nomogram−Predicted Probability of 12-month PFS in training cohort
n=433 d=337 p=11, 200 subjects per group
X − resampling optimism added, B=200
E
Actual 18-month PFS (proportion)
Nomogram−Predicted Probability of 18-month PFS in training cohort
n=433 d=337 p=11, 200 subjects per group
X − resampling optimism added, B=200
F
Actual 6-month PFS (proportion)
Nomogram−Predicted Probability of 6-month PFS in validation cohort
n=184 d=147 p=11, 80 subjects per group
X − resampling optimism added, B=200
G
Actual 12-month PFS (proportion)
Nomogram−Predicted Probability of 12-month PFS in validation cohort
n=184 d=147 p=11, 80 subjects per group
X − resampling optimism added, B=200
H
Actual 18-month PFS (proportion)
Nomogram−Predicted Probability of 18-month PFS in validation cohort
n=184 d=147 p=11, 80 subjects per group
X − resampling optimism added, B=200

Supplementary Figure 4. ROC curves and calibration curves for the PFS nomogram (A-B): ROC curve for the PFS nomogram in the training **(A)** and validation **(B)** cohorts; **(C-H):** Calibration curves for the 6-month **(C, F)**, 12-month **(D, G)**, and 18-month **(E, H)** PFS rates of the nomogram in the training cohort **(C-E)** and validation cohort **(F-H)**.

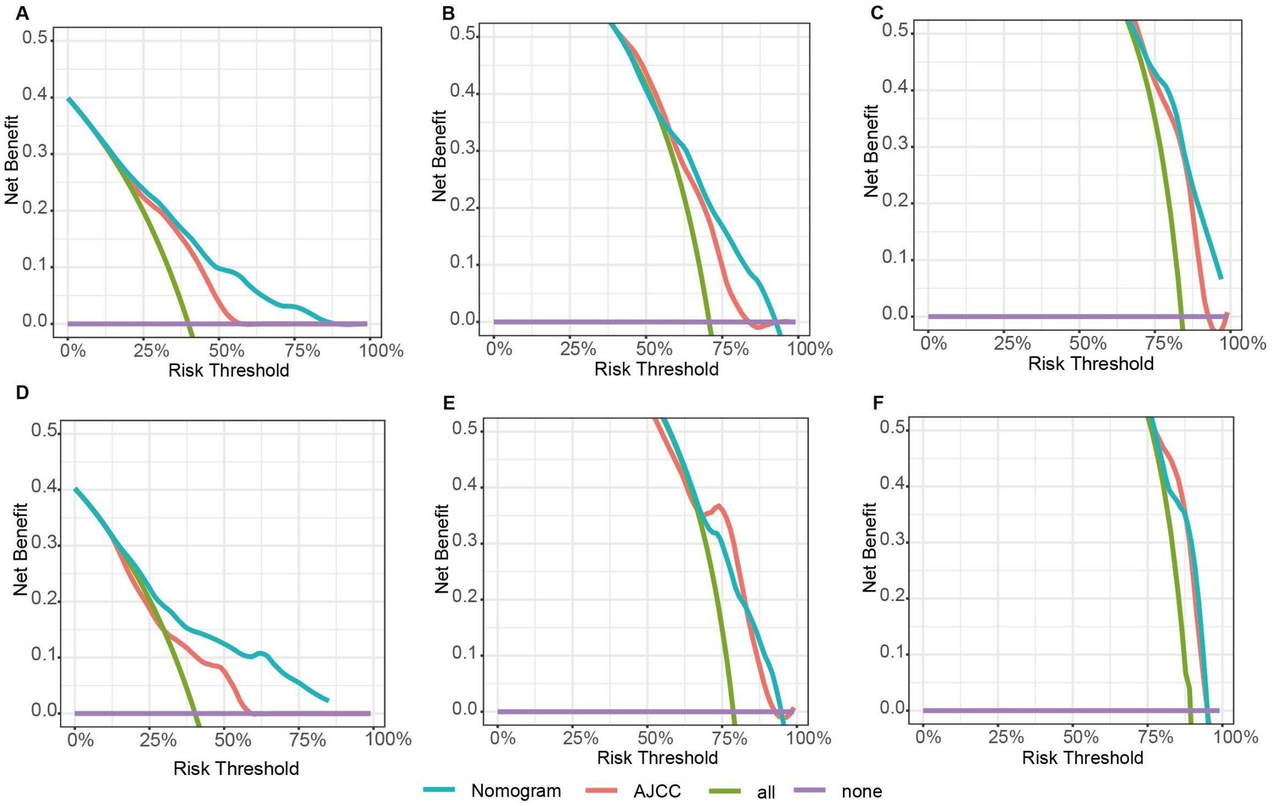

Supplementary Figure 5. DCA curves based on the nomogram model and AJCC tumor staging system (A-C): DCA curves for 6-month **(A)**, 12-month **(B)**, and 18-month **(C)** PFS improvement in the training cohort. **(D-F):** DCA curves for 6-month **(D)**, 12-month **(E)**, and 18-month **(F)** PFS improvement in the validation cohort.

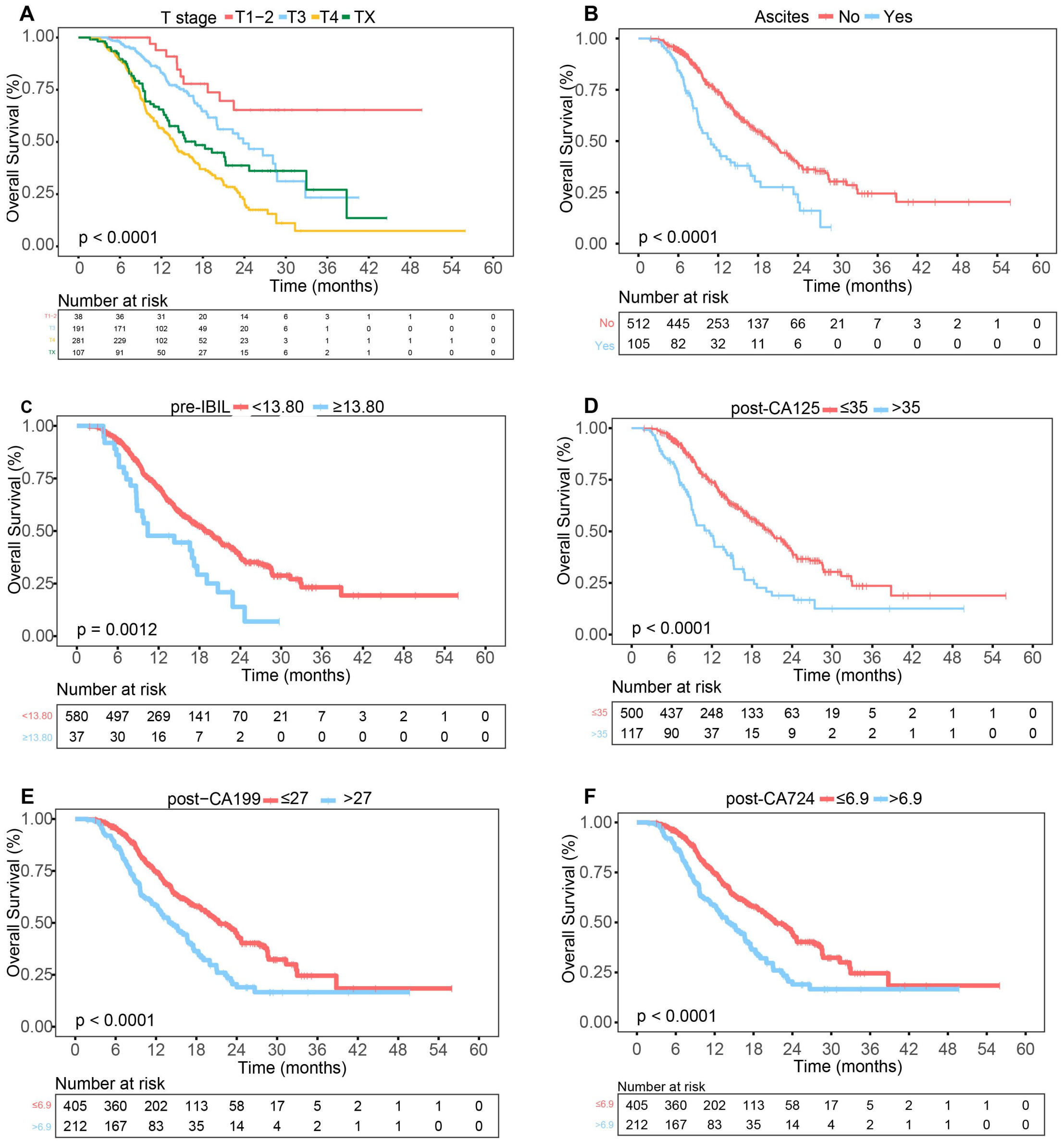

Supplementary Figure 6. OS Kaplan-Meier curves related to T stage **(A)**, ascites **(B)**, pre-IBIL **(C)**, post-CA125 **(D)**, post-CA199 **(E)**, and post-CA724 **(F)**.

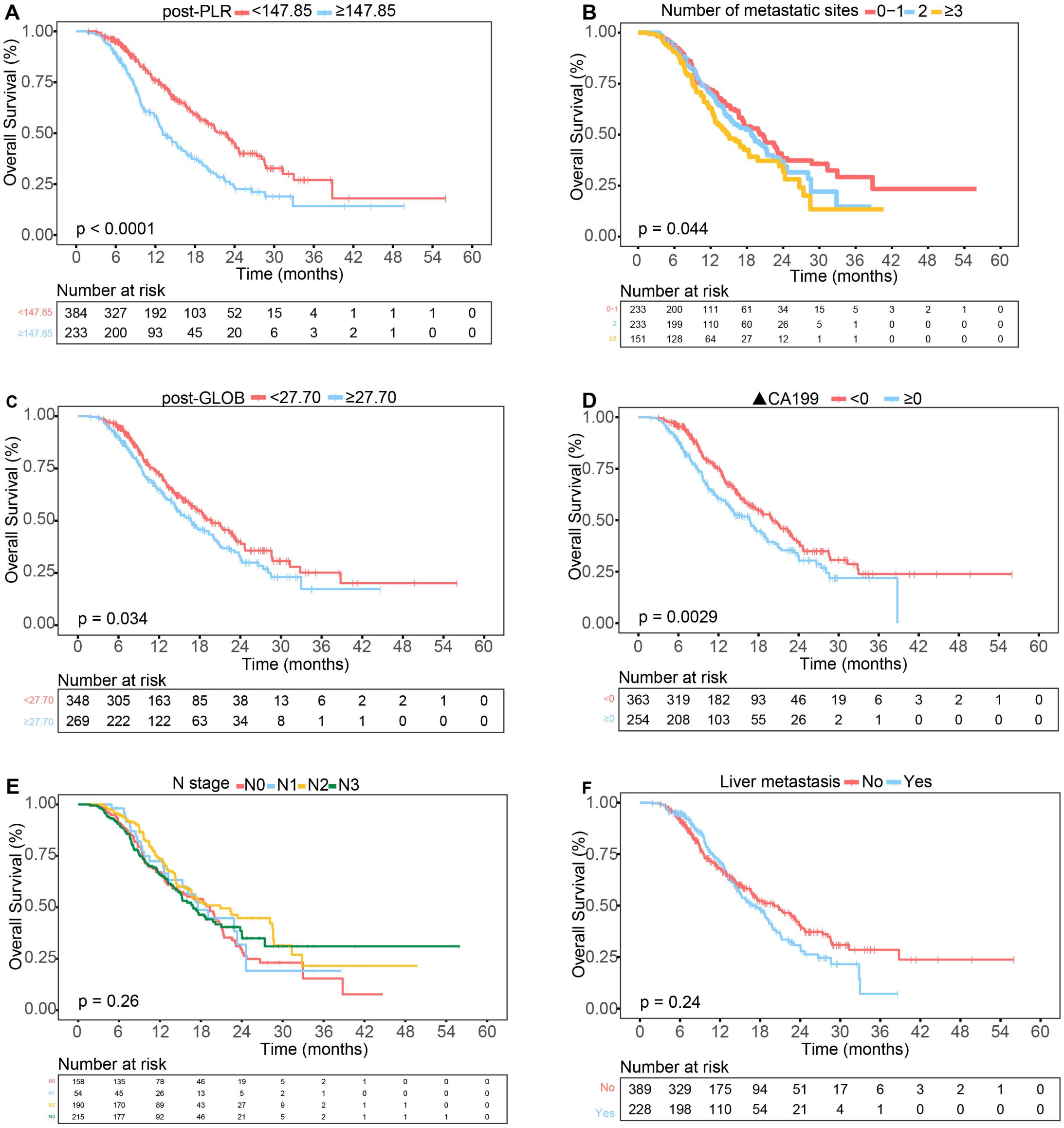

Supplementary Figure 7. OS Kaplan-Meier curves related to post-PLR **(A)**, number of metastatic sites **(B)**, post-

GLOB **(C)**, △CA199 **(D)**, N stage **(E)**, and liver metastasis

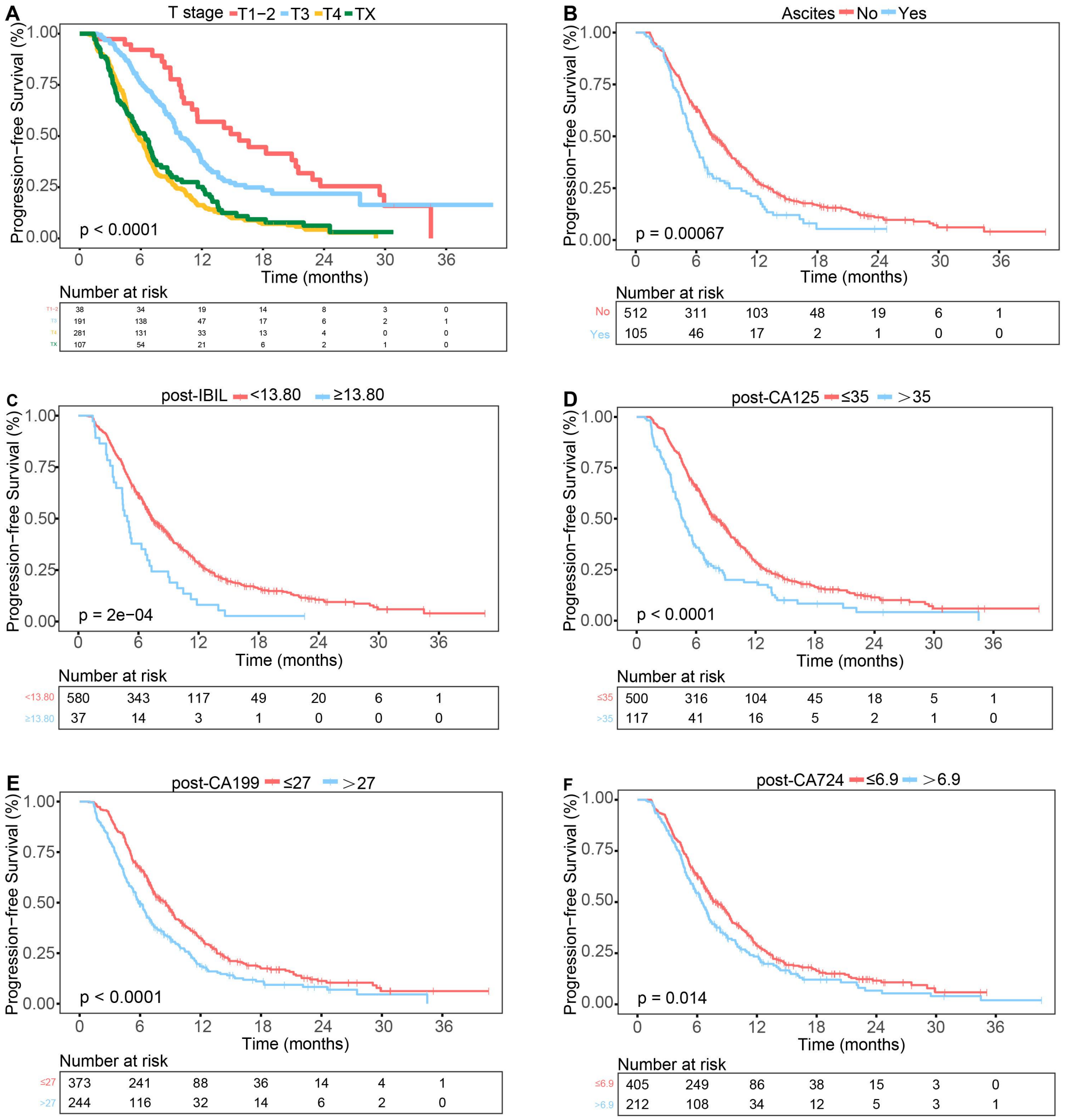

(F).

Supplementary Figure 8. PFS Kaplan-Meier curves related to T stage **(A)**, ascites **(B)**, pre-IBIL **(C)**, post-CA125 **(D)**, post-CA199 **(E)**, and post-CA724 **(F)**.

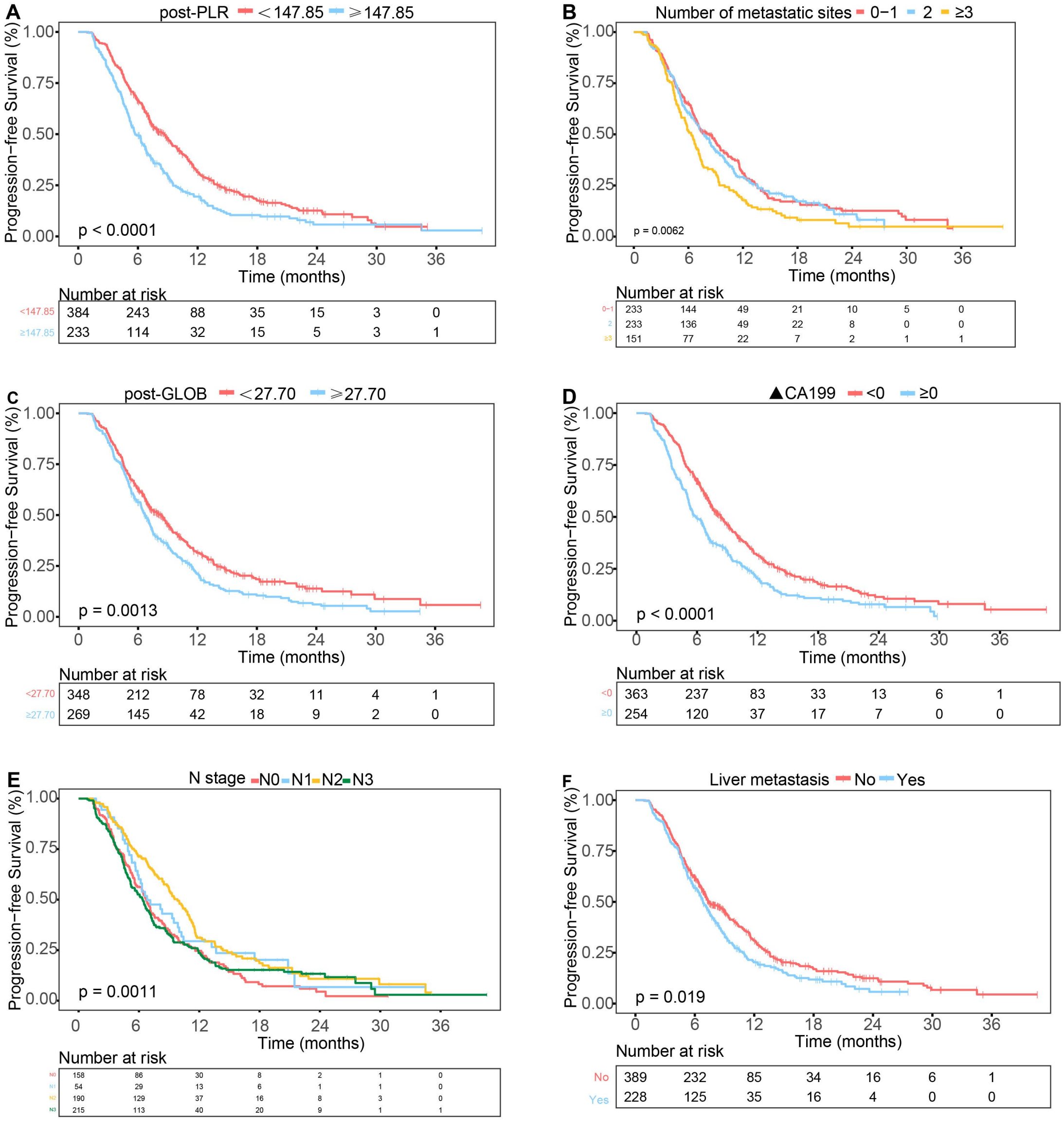

Supplementary Figure 9. PFS Kaplan-Meier curves related to post-PLR **(A)**, number of metastatic sites **(B)**, post-GLOB **(C)**, △CA199 **(D)**, N stage **(E)**, and liver metastasis **(F)**.

2 Supplementary Tables

Supplementary Table 1. Comparison of Baseline Characteristics Among CR+PR, SD, and PD Groups

Characteristics	CR+PR (n = 192)	SD (n = 364)	PD (n = 61)	*P* value
Gender				0.971
Male	133 (69.3%)	255 (70.1%)	42 (68.9%)	
Female	59 (30.7%)	109 (29.9%)	19 (31.1%)	
Age (years)				0.820
<50	23 (12%)	56 (15.4%)	7 (11.5%)	
50–59	61 (31.8%)	120 (33%)	23 (37.7%)	
60–69	66 (34.4%)	107 (29.4%)	18 (29.5%)	
≥70	42 (21.9%)	81 (22.3%)	13 (21.3%)	
Smoking history				0.654
No	130 (67.7%)	233 (64%)	41 (67.2%)	
Yes	62 (32.3%)	131 (36%)	20 (32.8%)	
Alcohol history				0.492
No	152 (79.2%)	273 (75%)	45 (73.8%)	
Yes	40 (20.8%)	91 (25%)	16 (26.2%)	
Agent				0.117
Sintilimab	96 (50%)	183 (50.3%)	30 (49.2%)	

Camrelizumab	52 (27.1%)	118 (32.4%)	14 (23%)	
Tislelizumab	12 (6.2%)	26 (7.1%)	6 (9.8%)	
Toripalimab	10 (5.2%)	8 (2.2%)	5 (8.2%)	
Penpulimab	5 (2.6%)	14 (3.8%)	3 (4.9%)	
Nivolumab	11 (5.7%)	6 (1.6%)	2 (3.3%)	
Pembrolizumab	6 (3.1%)	9 (2.5%)	1 (1.6%)	
Combination				<0.001
Chemotherapy	137 (71.4%)	265 (72.8%)	38 (62.3%)	
Targeted Therapy	4 (2.1%)	33 (9.1%)	11 (18%)	
Chemotherapy + Targeted Therapy	51 (26.6%)	66 (18.1%)	12 (19.7%)	
Treatment Line				<0.001
First-line	165 (85.9%)	262 (72%)	21 (34.4%)	
Second-line	23 (12%)	80 (22%)	29 (47.5%)	
Third-line and Later	4 (2.1%)	22 (6%)	11 (18%)	
Radical surgery				<0.001
No	172 (89.6%)	264 (72.5%)	43 (70.5%)	
Yes	20 (10.4%)	100 (27.5%)	18 (29.5%)	
Radiotherapy				0.259
No	189 (98.4%)	350 (96.2%)	60 (98.4%)	
Yes	3 (1.6%)	14 (3.8%)	1 (1.6%)	

BMI				0.044
Underweight（<18.5）	14 (7.3%)	53 (14.6%)	5 (8.2%)	
Normal（18.5-23.9）	102 (53.1%)	208 (57.1%)	39 (63.9%)	
Overweight（24-27.9）	57 (29.7%)	80 (22%)	13 (21.3%)	
Obese（≥28）	19 (9.9%)	23 (6.3%)	4 (6.6%)	
PD-L1 CPS				<0.001
CPS<1	32 (16.7%)	99 (27.2%)	9 (14.8%)	
CPS≥1	90 (46.9%)	111 (30.5%)	21 (34.4%)	
Unknown	70 (36.5%)	154 (42.3%)	31 (50.8%)	
Her-2				0.017
Negative	123 (64.1%)	264 (72.5%)	42 (68.9%)	
Positive	38 (19.8%)	44 (12.1%)	4 (6.6%)	
Unknown	31 (16.1%)	56 (15.4%)	15 (24.6%)	
ki-67				0.018
<70%	32 (16.7%)	100 (27.5%)	18 (29.5%)	
≥70%	89 (46.4%)	132 (36.3%)	18 (29.5%)	
Unknown	71 (37%)	132 (36.3%)	25 (41%)	
Pathological type				0.779
Adenocarcinoma	178 (92.7%)	343 (94.2%)	57 (93.4%)	

Others	14 (7.3%)	21 (5.8%)	4 (6.6%)	
Differentiation degree				0.675
Poorly	116 (60.4%)	213 (58.5%)	34 (55.7%)	
Moderately and Well	21 (10.9%)	52 (14.3%)	11 (18%)	
Unknown	55 (28.6%)	99 (27.2%)	16 (26.2%)	
Lauren classification				0.381
Intestinal type	19 (9.9%)	55 (15.1%)	7 (11.5%)	
Diffuse type	22 (11.5%)	49 (13.5%)	9 (14.8%)	
Mixed type	20 (10.4%)	39 (10.7%)	10 (16.4%)	
Unknown	131 (68.2%)	221 (60.7%)	35 (57.4%)	
Primary tumor site				0.588
Upper	108 (56.2%)	186 (51.1%)	32 (52.5%)	
Middle	44 (22.9%)	87 (23.9%)	13 (21.3%)	
Lower	38 (19.8%)	78 (21.4%)	15 (24.6%)	
Others	2 (1%)	13 (3.6%)	1 (1.6%)	
T stage				<0.001
T1-T2	16 (8.3%)	21 (5.8%)	1 (1.6%)	
T3	83 (43.2%)	103 (28.3%)	5 (8.2%)	
T4	76 (39.6%)	167 (45.9%)	38 (62.3%)	
TX	17 (8.9%)	73 (20.1%)	17 (27.9%)	
N stage				<0.001

N0	21 (10.9%)	116 (31.9%)	21 (34.4%)	
N1	19 (9.9%)	32 (8.8%)	3 (4.9%)	
N2	74 (38.5%)	107 (29.4%)	9 (14.8%)	
N3	78 (40.6%)	109 (29.9%)	28 (45.9%)	
M stage				0.242
M0	17 (8.9%)	45 (12.4%)	4 (6.6%)	
M1	175 (91.1%)	319 (87.6%)	57 (93.4%)	
Liver metastasis				<0.001
No	97 (50.5%)	256 (70.3%)	36 (59%)	
Yes	95 (49.5%)	108 (29.7%)	25 (41%)	
Bone metastasis				0.321
No	180 (93.8%)	334 (91.8%)	59 (96.7%)	
Yes	12 (6.2%)	30 (8.2%)	2 (3.3%)	
Lymph node metastasis				<0.001
No	12 (6.2%)	73 (20.1%)	14 (23%)	
Yes	180 (93.8%)	291 (79.9%)	47 (77%)	
Lung metastasis				0.122
No	169 (88%)	339 (93.1%)	55 (90.2%)	
Yes	23 (12%)	25 (6.9%)	6 (9.8%)	
Peritoneal metastasis				0.043

No	163 (84.9%)	277 (76.1%)	46 (75.4%)	
Yes	29 (15.1%)	87 (23.9%)	15 (24.6%)	
Ascites				0.003
No	174 (90.6%)	290 (79.7%)	48 (78.7%)	
Yes	18 (9.4%)	74 (20.3%)	13 (21.3%)	
Other metastases				0.181
No	165 (85.9%)	291 (79.9%)	48 (78.7%)	
Yes	27 (14.1%)	73 (20.1%)	13 (21.3%)	
Number of metastatic sites				0.227
0-1	63 (32.8%)	148 (40.7%)	22 (36.1%)	
2	84 (43.8%)	124 (34.1%)	25 (41%)	
≥3	45 (23.4%)	92 (25.3%)	14 (23%)	
pre-TP				0.923
<56.10	18 (9.4%)	38 (10.4%)	6 (9.8%)	
≥56.10	174 (90.6%)	326 (89.6%)	55 (90.2%)	
pre-ALB				0.828
<38.30	99 (51.6%)	180 (49.5%)	29 (47.5%)	
≥38.30	93 (48.4%)	184 (50.5%)	32 (52.5%)	
pre-GLOB				0.351
<22	30 (15.6%)	67 (18.4%)	7 (11.5%)	

≥22	162 (84.4%)	297 (81.6%)	54 (88.5%)	
pre-TBIL				0.007
<8.70	91 (47.4%)	149 (40.9%)	15 (24.6%)	
≥8.70	101 (52.6%)	215 (59.1%)	46 (75.4%)	
pre-DBIL				0.192
<4.30	119 (62%)	208 (57.1%)	30 (49.2%)	
≥4.30	73 (38%)	156 (42.9%)	31 (50.8%)	
pre-IBIL				0.017
<13.80	188 (97.9%)	337 (92.6%)	55 (90.2%)	
≥13.80	4 (2.1%)	27 (7.4%)	6 (9.8%)	
pre-CA125				0.085
≤35	126 (65.6%)	254 (69.8%)	34 (55.7%)	
>35	66 (34.4%)	110 (30.2%)	27 (44.3%)	
pre-CA199				0.119
≤27	105 (54.7%)	200 (54.9%)	25 (41%)	
>27	87 (45.3%)	164 (45.1%)	36 (59%)	
pre-CA724				0.697
≤6.9	108 (56.2%)	218 (59.9%)	35 (57.4%)	
>6.9	84 (43.8%)	146 (40.1%)	26 (42.6%)	
pre-CEA				<0.001

≤4.7	82 (42.7%)	201 (55.2%)	21 (34.4%)	
>4.7	110 (57.3%)	163 (44.8%)	40 (65.6%)	
pre-PNI				0.469
<45.25	90 (46.9%)	182 (50%)	34 (55.7%)	
≥45.25	102 (53.1%)	182 (50%)	27 (44.3%)	
pre-NLR				0.977
<4.25	144 (75%)	276 (75.8%)	46 (75.4%)	
≥4.25	48 (25%)	88 (24.2%)	15 (24.6%)	
pre-PLR				0.484
<174.44	104 (54.2%)	216 (59.3%)	34 (55.7%)	
≥174.44	88 (45.8%)	148 (40.7%)	27 (44.3%)	
pre-MLR				0.110
<0.36	113 (58.9%)	233 (64%)	31 (50.8%)	
≥0.36	79 (41.1%)	131 (36%)	30 (49.2%)	
pre-NMR				0.081
<29.91	188 (97.9%)	351 (96.4%)	56 (91.8%)	
≥29.91	4 (2.1%)	13 (3.6%)	5 (8.2%)	
pre-SII				0.242
<796.93	114 (59.4%)	240 (65.9%)	36 (59%)	
≥796.93	78 (40.6%)	124 (34.1%)	25 (41%)	
pre-NLPR				0.208

<0.02	126 (65.6%)	218 (59.9%)	33 (54.1%)	
≥0.02	66 (34.4%)	146 (40.1%)	28 (45.9%)	
pre-AISI				0.468
<681.35	162 (84.4%)	309 (84.9%)	48 (78.7%)	
≥681.35	30 (15.6%)	55 (15.1%)	13 (21.3%)	
pre-SIRI				0.493
<1.91	140 (72.9%)	271 (74.5%)	41 (67.2%)	
≥1.91	52 (27.1%)	93 (25.5%)	20 (32.8%)	
pre-RAR				0.035
<0.40	122 (63.5%)	197 (54.1%)	29 (47.5%)	
≥0.40	70 (36.5%)	167 (45.9%)	32 (52.5%)	
pre-RPR				<0.001
<0.07	117 (60.9%)	148 (40.7%)	20 (32.8%)	
≥0.07	75 (39.1%)	216 (59.3%)	41 (67.2%)	
pre-RLR				<0.001
<15	152 (79.2%)	263 (72.3%)	33 (54.1%)	
≥15	40 (20.8%)	101 (27.7%)	28 (45.9%)	
pre-HPR				0.026
<0.66	146 (76%)	240 (65.9%)	38 (62.3%)	
≥0.66	46 (24%)	124 (34.1%)	23 (37.7%)	

post-TP				0.604
<78.70	187 (97.4%)	358 (98.4%)	59 (96.7%)	
≥78.70	5 (2.6%)	6 (1.6%)	2 (3.3%)	
post-ALB				0.469
<38.80	89 (46.4%)	185 (50.8%)	33 (54.1%)	
≥38.80	103 (53.6%)	179 (49.2%)	28 (45.9%)	
post-GLOB				0.100
<27.70	115 (59.9%)	206 (56.6%)	27 (44.3%)	
≥27.70	77 (40.1%)	158 (43.4%)	34 (55.7%)	
post-TBIL				0.110
<9.90	102 (53.1%)	178 (48.9%)	23 (37.7%)	
≥9.90	90 (46.9%)	186 (51.1%)	38 (62.3%)	
post-DBIL				0.067
<6.35	166 (86.5%)	304 (83.5%)	45 (73.8%)	
≥6.35	26 (13.5%)	60 (16.5%)	16 (26.2%)	
post-IBIL				0.510
<9.73	162 (84.4%)	293 (80.5%)	49 (80.3%)	
≥9.73	30 (15.6%)	71 (19.5%)	12 (19.7%)	
post-CA125				<0.001
≤35	171 (89.1%)	294 (80.8%)	35 (57.4%)	
>35	21 (10.9%)	70 (19.2%)	26 (42.6%)	

post-CA199				<0.001
≤27	130 (67.7%)	219 (60.2%)	24 (39.3%)	
>27	62 (32.3%)	145 (39.8%)	37 (60.7%)	
post-CA724				0.222
≤6.9	133 (69.3%)	237 (65.1%)	35 (57.4%)	
>6.9	59 (30.7%)	127 (34.9%)	26 (42.6%)	
post-CEA				<0.001
≤4.7	105 (54.7%)	207 (56.9%)	19 (31.1%)	
>4.7	87 (45.3%)	157 (43.1%)	42 (68.9%)	
post-PNI				0.026
<45.85	83 (43.2%)	185 (50.8%)	38 (62.3%)	
≥45.85	109 (56.8%)	179 (49.2%)	23 (37.7%)	
post-NLR				<0.001
<2.21	127 (66.1%)	205 (56.3%)	21 (34.4%)	
≥2.21	65 (33.9%)	159 (43.7%)	40 (65.6%)	
post-PLR				0.001
<147.85	132 (68.8%)	226 (62.1%)	26 (42.6%)	
≥147.85	60 (31.2%)	138 (37.9%)	35 (57.4%)	
post-MLR				0.410
<0.30	93 (48.4%)	157 (43.1%)	25 (41%)	

≥0.30	99 (51.6%)	207 (56.9%)	36 (59%)	
post-NMR				0.003
<6.50	105 (54.7%)	172 (47.3%)	18 (29.5%)	
≥6.50	87 (45.3%)	192 (52.7%)	43 (70.5%)	
post-SII				0.003
<301.30	89 (46.4%)	163 (44.8%)	14 (23%)	
≥301.30	103 (53.6%)	201 (55.2%)	47 (77%)	
post-NLPR				0.012
<0.05	190 (99%)	342 (94%)	56 (91.8%)	
≥0.05	2 (1%)	22 (6%)	5 (8.2%)	
post-AISI				0.131
<76.86	62 (32.3%)	98 (26.9%)	12 (19.7%)	
≥76.86	130 (67.7%)	266 (73.1%)	49 (80.3%)	
post-SIRI				0.416
<0.65	77 (40.1%)	152 (41.8%)	20 (32.8%)	
≥0.65	115 (59.9%)	212 (58.2%)	41 (67.2%)	
post-RAR				0.963
<0.45	91 (47.4%)	176 (48.4%)	30 (49.2%)	
≥0.45	101 (52.6%)	188 (51.6%)	31 (50.8%)	
post-RPR				0.315
<0.39	189 (98.4%)	354 (97.3%)	61 (100%)	

≥0.39	3 (1.6%)	10 (2.7%)	0 (0%)	
post-RLR				0.105
<17.02	146 (76%)	248 (68.1%)	40 (65.6%)	
≥17.02	46 (24%)	116 (31.9%)	21 (34.4%)	
post-HPR				0.678
<1.39	182 (94.8%)	338 (92.9%)	57 (93.4%)	
≥1.39	10 (5.2%)	26 (7.1%)	4 (6.6%)	
△CA125				<0.001
<0	146 (76%)	235 (64.6%)	28 (45.9%)	
≥0	46 (24%)	129 (35.4%)	33 (54.1%)	
△CA199				<0.001
<0	132 (68.8%)	205 (56.3%)	26 (42.6%)	
≥0	60 (31.2%)	159 (43.7%)	35 (57.4%)	
△CA724				<0.001
<0	142 (74%)	206 (56.6%)	33 (54.1%)	
≥0	50 (26%)	158 (43.4%)	28 (45.9%)	
△CEA				<0.001
<0	128 (66.7%)	183 (50.3%)	25 (41%)	
≥0	64 (33.3%)	181 (49.7%)	36 (59%)	
△PNI				0.826

<0	92 (47.9%)	178 (48.9%)	32 (52.5%)	
≥0	100 (52.1%)	186 (51.1%)	29 (47.5%)	
△NLR				<0.001
<0	160 (83.3%)	241 (66.2%)	35 (57.4%)	
≥0	32 (16.7%)	123 (33.8%)	26 (42.6%)	
△PLR				<0.001
<0	148 (77.1%)	242 (66.5%)	27 (44.3%)	
≥0	44 (22.9%)	122 (33.5%)	34 (55.7%)	
△MLR				0.420
<0	98 (51%)	166 (45.6%)	31 (50.8%)	
≥0	94 (49%)	198 (54.4%)	30 (49.2%)	
△NMR				<0.001
<0	158 (82.3%)	245 (67.3%)	38 (62.3%)	
≥0	34 (17.7%)	119 (32.7%)	23 (37.7%)	
△SII				<0.001
<0	157 (81.8%)	256 (70.3%)	35 (57.4%)	
≥0	35 (18.2%)	108 (29.7%)	26 (42.6%)	
△NLPR				0.641
<0	51 (26.6%)	102 (28%)	20 (32.8%)	
≥0	141 (73.4%)	262 (72%)	41 (67.2%)	
△AISI				<0.001

<0	154 (80.2%)	241 (66.2%)	34 (55.7%)	
≥0	38 (19.8%)	123 (33.8%)	27 (44.3%)	
△SIRI				0.004
<0	140 (72.9%)	221 (60.7%)	33 (54.1%)	
≥0	52 (27.1%)	143 (39.3%)	28 (45.9%)	
△RAR				<0.001
<0	32 (16.7%)	99 (27.2%)	24 (39.3%)	
≥0	160 (83.3%)	265 (72.8%)	37 (60.7%)	
△RPR				<0.001
<0	27 (14.1%)	78 (21.4%)	22 (36.1%)	
≥0	165 (85.9%)	286 (78.6%)	39 (63.9%)	
△RLR				0.171
<0	65 (33.9%)	123 (33.8%)	28 (45.9%)	
≥0	127 (66.1%)	241 (66.2%)	33 (54.1%)	
△HPR				<0.001
<0	39 (20.3%)	123 (33.8%)	28 (45.9%)	
≥0	153 (79.7%)	241 (66.2%)	33 (54.1%)	

Supplementary Table 2. Efficacy Evaluation in High and Low PD-L1 Expression Groups

	High PD-L1 Expression (n = 222)	Low PD-L1 Expression (n = 140)	*P* value
CR	1 (0.5%)	0 (0%)	0.001
PR	89 (40.1%)	32 (22.9%)	
SD	111 (50%)	99 (70.7%)	
PD	21 (9.5%)	9 (6.4%)	
ORR	40.54%	22.86%	
DCR	90.54%	93.57%	
mOS	21.00 months (95%CI: 15.72 - 26.29)	17.83 months (95%CI: 14.88 - 20.78)	
mPFS	8.13 months (95%CI: 6.96 - 9.30)	7.17 months (95%CI: 6.31 - 8.03)	

Supplementary Table 3. Efficacy Evaluation in Her-2 Positive and Negative Groups

	Her-2 Positive (n = 86)	Her-2 Negative (n = 429)	*P* value
CR	1 (0.5%)	0 (0%)	0.004
PR	37 (43%)	123 (28.7%)	
SD	44 (51.2%)	264 (61.5%)	
PD	4 (4.7%)	42 (9.8%)	
ORR	44.19%	28.67%	
DCR	95.35%	90.21%	
mOS	22.87 months (95%CI：19.66 -	17.20 months (95%CI: 14.97 - 19.43)	

	26.08)	
mPFS	8.93 months (95%CI: 5.95 - 11.91)	6.97 months (95%CI: 6.41 - 7.53)

Supplementary Table 4. Efficacy Evaluation in Groups with Different Treatment Lines

	First-line Treatment (n = 448)	Second-line Treatment (n = 132)	Third-line and Later Treatment (n = 37)	*P* value
CR	0 (0%)	1 (0.8%)	0 (0%)	
PR	165 (36.8%)	22 (16.7%)	4 (10.8%)	<0.001
SD	262 (58.5%)	80 (60.6%)	22 (59.5%)	
PD	21 (4.7%)	29 (22%)	11 (29.7%)	
ORR	36.83%	17.42%	10.81%	
DCR	95.31%	78.03%	70.27%	
mOS	20.97 months (95%CI: 18.15 - 23.79)	12.77 months (95%CI: 10.62 - 14.92)	15.47 months (95%CI: 9.04 - 21.90)	
mPFS	8.33 months (95%CI: 7.46 - 9.20)	5.17 months (95%CI: 3.98 - 6.36)	4.77 months (95%CI: 2.70 - 6.84)	

Supplementary Table 5. Efficacy Evaluation of Patients in First-Line Treatment

	PD-L1 CPS ≥1 (n=100)	PD-L1 CPS <1 (n=171)	Her-2 Positive (n=59)	Her-2 Negative (n=316)	Immunotherapy + Chemotherapy (n=356)	Immunotherapy + Targeted Therapy (n=12)	Immunotherapy + Chemotherapy + Targeted Therapy (n=80)

CR	0	0	0	0	0	0	0
PR	27	81	32	106	124	2	39
SD	71	83	26	195	214	9	39
PD	2	7	1	15	18	1	2
ORR	27.00%	47.37%	54.24%	33.54%	34.83%	16.67%	48.75%
DCR	98.00%	95.91%	98.31%	95.25%	94.94%	91.67%	97.50%
mOS	23.83 months (95%CI: 20.97 - 26.69)	18.77 months (95%CI: 16.13 - 21.41)	28.13 months (95%CI: 20.47 - 35.80)	18.77 months (95%CI: 15.83 - 21.71)	19.07 months (95%CI: 16.49 - 21.65)	21.23 months (95%CI: 0.00 - 47.58)	28.13 months (95%CI: 22.17 - 34.10)
mPFS	8.80 months (95%CI: 7.88 - 9.72)	8.00 months (95%CI: 6.10 - 9.90)	11.60 months (95%CI: 9.52 - 13.68)	7.67 months (95%CI: 6.73 - 8.61)	7.97 months (95%CI: 6.99 - 8.95)	6.73 months (95%CI: 3.84 - 9.62)	9.47 months (95%CI: 8.11 - 10.83)

Supplementary Table 6. Efficacy Evaluation by PD-L1 Stratification in Her-2 Negative Patients Receiving First-line Immunotherapy Combined with Chemotherapy

	PD-L1 CPS⩾1 (n = 131)	PD-L1 CPS<1 (n = 74)	*P* value
CR	0 (0%)	0 (0%)	
PR	56 (42.75%)	18 (24.32%)	0.017
SD	69 (52.67%)	54 (72.97%)	

PD	6 (4.58%)	2 (2.70%)
ORR	42.75%	24.32%
DCR	95.42%	97.30%
mOS	20.87 months	18.77 months
mPFS	7.97 months	8.00 months

Supplementary Table 7. C-Index, NRIs, and IDIs for Predicting OS Using the Nomogram and AJCC Criteria

Metric	Training Cohort			Validation Cohort		
	Estimate	95% CI	*P* value	Estimate	95% CI	*P* value
C-Index						
Nomogram: OS	0.728	(0.689 - 0.767)		0.742	(0.681 - 0.803)	
AJCC：OS	0.641	(0.600 - 0.682)		0.679	(0.618 - 0.740)	
NRI (vs AJCC)						
12-month OS	0.110	(0.062 - 0.159)	<0.001	0.157	(0.062 - 0.252)	0.001
18-month OS	0.211	(0.120 - 0.303)	<0.001	0.133	(-0.021 - 0.287)	0.091
24-month OS	0.121	(0.020 - 0.223)	0.019	0.025	(-0.130 - 0.180)	0.752
IDI (vs AJCC)						
12-month OS	0.097	(0.067 - 0.126)	0	0.085	(0.030 - 0.141)	0.003
18-month OS	0.108	(0.075 - 0.142)	0	0.070	(0.009 - 0.131)	0.025
24-month OS	0.102	(0.069 - 0.134)	0	0.041	(-0.015 - 0.097)	0.149

Supplementary Table 8. C-Index, NRIs, and IDIs for Predicting PFS Using the Nomogram and AJCC Staging System

Metric	Training Cohort			Validation Cohort		
	Estimate	95% CI	*P* value	Estimate	95% CI	*P* value
C-Index						
Nomogram: PFS	0.695	(0.668 - 0.722)		0.687	(0.636 - 0.738)	
AJCC：PFS	0.648	(0.619 - 0.677)		0.637	(0.584 - 0.690)	
NRI (vs AJCC)						
6-month PFS	0.097	(-0.005 - 0.199)	0.063	0.068	(-0.084 - 0.220)	0.381
12-month PFS	0.073	(-0.031 - 0.178)	0.17	0.230	(0.086 - 0.373)	0.002
18-month PFS	0.015	(-0.029 - 0.059)	0.500	0.034	(-0.043 - 0.110)	0.388
IDI (vs AJCC)						
6-month PFS	0.049	(0.026 - 0.072)	<0.001	0.086	(0.034 - 0.139)	0.001
12-month PFS	0.033	(0.007 - 0.058)	0.012	0.073	(0.016 - 0.129)	0.012
18-month PFS	0.016	(-0.006 - 0.038)	0.157	0.061	(0.012 - 0.109)	0.014

Supplementary Table 9. Characteristics of Patients with Different N Stages

Characteristic	N0 (n = 158)	N1 (n = 54)	N2 (n = 190)	N3 (n = 215)	*P* value
Gender					0.611
Male	115 (72.8%)	38 (70.4%)	134 (70.5%)	143 (66.5%)	
Female	43 (27.2%)	16 (29.6%)	56 (29.5%)	72 (33.5%)	

Age (years)					0.026
<50	20 (12.7%)	4 (7.4%)	20 (10.5%)	42 (19.5%)	
50–59	55 (34.8%)	19 (35.2%)	52 (27.4%)	78 (36.3%)	
60–69	49 (31%)	19 (35.2%)	64 (33.7%)	59 (27.4%)	
≥70	34 (21.5%)	12 (22.2%)	54 (28.4%)	36 (16.7%)	
Smoking history					0.310
No	102 (64.6%)	37 (68.5%)	133 (70%)	132 (61.4%)	
Yes	56 (35.4%)	17 (31.5%)	57 (30%)	83 (38.6%)	
Alcohol history					0.275
No	123 (77.8%)	40 (74.1%)	152 (80%)	155 (72.1%)	
Yes	35 (22.2%)	14 (25.9%)	38 (20%)	60 (27.9%)	
Agent					0.029
Sintilimab	83 (52.5%)	27 (50%)	99 (52.1%)	100 (46.5%)	
Camrelizumab	53 (33.5%)	14 (25.9%)	59 (31.1%)	58 (27%)	
Tislelizumab	11 (7%)	3 (5.6%)	12 (6.3%)	18 (8.4%)	
Toripalimab	3 (1.9%)	4 (7.4%)	6 (3.2%)	10 (4.7%)	
Penpulimab	4 (2.5%)	4 (7.4%)	9 (4.7%)	5 (2.3%)	
Nivolumab	2 (1.3%)	2 (3.7%)	3 (1.6%)	12 (5.6%)	
Pembrolizumab	2 (1.3%)	0 (0%)	2 (1.1%)	12 (5.6%)	
Combination					<0.001

Chemotherapy	107 (67.7%)	38 (70.4%)	150 (78.9%)	145 (67.4%)	
Targeted Therapy	23 (14.6%)	6 (11.1%)	8 (4.2%)	11 (5.1%)	
Chemotherapy + Targeted Therapy	28 (17.7%)	10 (18.5%)	32 (16.8%)	59 (27.4%)	
Treatment Line					0.003
First-line	103 (65.2%)	37 (68.5%)	156 (82.1%)	152 (70.7%)	
Second-line	38 (24.1%)	15 (27.8%)	30 (15.8%)	49 (22.8%)	
Third-line and Later	17 (10.8%)	2 (3.7%)	4 (2.1%)	14 (6.5%)	
Radical surgery					<0.001
No	61 (38.6%)	46 (85.2%)	177 (93.2%)	195 (90.7%)	
Yes	97 (61.4%)	8 (14.8%)	13 (6.8%)	20 (9.3%)	
Radiotherapy					0.003
No	148 (93.7%)	51 (94.4%)	190 (100%)	210 (97.7%)	
Yes	10 (6.3%)	3 (5.6%)	0 (0%)	5 (2.3%)	
BMI					0.002
Underweight（<18.5）	31 (19.6%)	5 (9.3%)	21 (11.1%)	15 (7%)	
Normal（18.5-23.9）	96 (60.8%)	30 (55.6%)	96 (50.5%)	127 (59.1%)	
Overweight（24-27.9）	25 (15.8%)	15 (27.8%)	55 (28.9%)	55 (25.6%)	
Obese（≥28）	6 (3.8%)	4 (7.4%)	18 (9.5%)	18 (8.4%)	
PD-L1 CPS					0.001

CPS<1	42 (26.6%)	11 (20.4%)	35 (18.4%)	52 (24.2%)	
CPS≥1	39 (24.7%)	16 (29.6%)	72 (37.9%)	95 (44.2%)	
Unknown	77 (48.7%)	27 (50%)	83 (43.7%)	68 (31.6%)	
Her-2					0.014
Negative	119 (75.3%)	39 (72.2%)	127 (66.8%)	144 (67%)	
Positive	19 (12%)	2 (3.7%)	24 (12.6%)	41 (19.1%)	
Unknown	20 (12.7%)	13 (24.1%)	39 (20.5%)	30 (14%)	
ki-67					0.005
<70%	56 (35.4%)	12 (22.2%)	40 (21.1%)	42 (19.5%)	
≥70%	55 (34.8%)	27 (50%)	72 (37.9%)	85 (39.5%)	
Unknown	47 (29.7%)	15 (27.8%)	78 (41.1%)	88 (40.9%)	
Pathological type					0.119
Adenocarcinoma	149 (94.3%)	50 (92.6%)	172 (90.5%)	207 (96.3%)	
Others	9 (5.7%)	4 (7.4%)	18 (9.5%)	8 (3.7%)	
Differentiation degree					0.049
Poorly	102 (64.6%)	31 (57.4%)	99 (52.1%)	131 (60.9%)	
Moderately and Well	24 (15.2%)	8 (14.8%)	33 (17.4%)	19 (8.8%)	
Unknown	32 (20.3%)	15 (27.8%)	58 (30.5%)	65 (30.2%)	
Lauren classification					0.004
Intestinal type	26 (16.5%)	7 (13%)	25 (13.2%)	23 (10.7%)	

Diffuse type	27 (17.1%)	9 (16.7%)	21 (11.1%)	23 (10.7%)	
Mixed type	29 (18.4%)	4 (7.4%)	18 (9.5%)	18 (8.4%)	
Unknown	76 (48.1%)	34 (63%)	126 (66.3%)	151 (70.2%)	
Primary tumor site					0.002
Upper	84 (53.2%)	41 (75.9%)	102 (53.7%)	99 (46%)	
Middle	29 (18.4%)	8 (14.8%)	49 (25.8%)	58 (27%)	
Lower	36 (22.8%)	5 (9.3%)	37 (19.5%）	53 (24.7%)	
Others	9 (5.7%)	0 (0%)	2 (1.1%)	5 (2.3%)	
T stage					<0.001
T1-T2	11 (7%)	3 (5.6%)	20 (10.5%)	4 (1.9%)	
T3	25 (15.8%)	28 (51.9%)	82 (43.2%)	56 (26%)	
T4	44 (27.8%)	18 (33.3%)	79 (41.6%)	140 (65.1%)	
TX	78 (49.4%)	5 (9.3%)	9 (4.7%)	15 (7%)	
M stage					0.003
M0	12 (7.6%)	9 (16.7%)	31 (16.3%)	14 (6.5%)	
M1	146 (92.4%)	45 (83.3%)	159 (83.7%)	201 (93.5%)	
Liver metastasis					0.119
No	102 (64.6%)	27 (50%)	116 (61.1%)	144 (67%)	
Yes	56 (35.4%)	27 (50%)	74 (38.9%)	71 (33%)	
Bone metastasis					0.065
No	143 (90.5%)	52 (96.3%)	183 (96.3%)	195 (90.7%)	

Yes	15 (9.5%)	2 (3.7%)	7 (3.7%)	20 (9.3%)	
Lymph node metastasis					<0.001
No	99 (62.7%)	0 (0%)	0 (0%)	0 (0%)	
Yes	59 (37.3%)	54 (100%)	190 (100%)	215 (100%)	
Lung metastasis					0.420
No	145 (91.8%)	52 (96.3%)	174 (91.6%)	192 (89.3%)	
Yes	13 (8.2%)	2 (3.7%)	16 (8.4%)	23 (10.7%)	
Peritoneal metastasis					<0.001
No	128 (81%)	47 (87%)	162 (85.3%)	149 (69.3%)	
Yes	30 (19%)	7 (13%)	28 (14.7%)	66 (30.7%)	
Ascites					<0.001
No	129 (81.6%)	47 (87%)	174 (91.6%)	162 (75.3%)	
Yes	29 (18.4%)	7 (13%)	16 (8.4%)	53 (24.7%)	
Other metastases					<0.001
No	113 (71.5%)	48 (88.9%)	169 (88.9%)	174 (80.9%)	
Yes	45 (28.5%)	6 (11.1%)	21 (11.1%)	41 (19.1%)	
Number of metastatic sites					<0.001
0-1	84 (53.2%)	17 (31.5%)	73 (38.4%)	59 (27.4%)	
2	53 (33.5%)	26 (48.1%)	84 (44.2%)	70 (32.6%)	

≥3	21 (13.3%)	11 (20.4%)	33 (17.4%)	86 (40%)

Supplementary Table 10. Characteristics of Patients With and Without Liver Metastasis

Characteristic	No Liver Metastasis（n = 389）	Liver Metastasis（n = 228）	*P* value
Gender			0.231
Male	264 (67.9%)	166 (72.8%)	
Female	125 (32.1%)	62 (27.2%)	
Age (years)			0.003
<50	69 (17.7%)	17 (7.5%)	
50–59	123 (31.6%)	81 (35.5%)	
60–69	110 (28.3%)	81 (35.5%)	
≥70	87 (22.4%)	49 (21.5%)	
Smoking history			0.625
No	258 (66.3%)	146 (64%)	
Yes	131 (33.7%)	82 (36%)	
Alcohol history			0.226
No	303 (77.9%)	167 (73.2%)	
Yes	86 (22.1%)	61 (26.8%)	
Agent			0.193

Sintilimab	201 (51.7%)	108 (47.4%)	
Camrelizumab	113 (29%)	71 (31.1%)	
Tislelizumab	32 (8.2%)	12 (5.3%)	
Toripalimab	10 (2.6%)	13 (5.7%)	
Penpulimab	13 (3.3%)	9 (3.9%)	
Nivolumab	9 (2.3%)	10 (4.4%)	
Pembrolizumab	11 (2.8%)	5 (2.2%)	
Combination			0.066
Chemotherapy	287 (73.8%)	153 (67.1%)	
Targeted Therapy	32 (8.2%)	16 (7%)	
Chemotherapy + Targeted Therapy	70 (18%)	59 (25.9%)	
Treatment Line			0.036
First-line	289 (74.3%)	159 (69.7%)	
Second-line	84 (21.6%)	48 (21.1%)	
Third-line and Later	16 (4.1%)	21 (9.2%)	
Radical surgery			0.036
No	291 (74.8%)	188 (82.5%)	
Yes	98 (25.2%)	40 (17.5%)	
Radiotherapy			0.118
No	374 (96.1%)	225 (98.7%)	

Yes	15 (3.9%)	3 (1.3%)	
BMI			0.036
Underweight（<18.5）	55 (14.1%)	17 (7.5%)	
Normal（18.5-23.9）	222 (57.1%)	127 (55.7%)	
Overweight（24-27.9）	85 (21.9%)	65 (28.5%)	
Obese（≥28）	27 (6.9%)	19 (8.3%)	
PD-L1 CPS			0.666
CPS<1	87 (22.4%)	53 (23.2%)	
CPS≥1	136 (35%)	86 (37.7%)	
Unknown	166 (42.7%)	89 (39%)	
Her-2			0.421
Negative	273 (70.2%)	156 (68.4%)	
Positive	49 (12.6%)	37 (16.2%)	
Unknown	67 (17.2%)	35 (15.4%)	
ki-67			0.004
<70%	110 (28.3%)	40 (17.5%)	
≥70%	135 (34.7%)	104 (45.6%)	
Unknown	144 (37%)	84 (36.8%)	
Pathological type			0.002
Adenocarcinoma	374 (96.1%)	204 (89.5%)	

Others	15 (3.9%)	24 (10.5%)	
Differentiation degree			0.005
Poorly	242 (62.2%)	121 (53.1%)	
Moderately and Well	40 (10.3%)	44 (19.3%)	
Unknown	107 (27.5%)	63 (27.6%)	
Lauren classification			<0.001
Intestinal type	37 (9.5%)	44 (19.3%)	
Diffuse type	66 (17%)	14 (6.1%)	
Mixed type	44 (11.3%)	25 (11%)	
Unknown	242 (62.2%)	145 (63.6%)	
Primary tumor site			0.211
Upper	197 (50.6%)	129 (56.6%)	
Middle	101 (26%)	43 (18.9%)	
Lower	80 (20.6%)	51 (22.4%)	
Others	11 (2.8%)	5 (2.2%)	
T stage			0.186
T1-T2	22 (5.7%)	16 (7%)	
T3	115 (29.6%)	76 (33.3%)	
T4	175 (45%)	106 (46.5%)	
TX	77 (19.8%)	30 (13.2%)	

N stage			0.119
N0	102 (26.2%)	56 (24.6%)	
N1	27 (6.9%)	27 (11.8%)	
N2	116 (29.8%)	74 (32.5%)	
N3	144 (37%)	71 (31.1%)	
M stage			<0.001
M0	66 (17%)	0 (0%)	
M1	323 (83%)	228 (100%)	
Bone metastasis			0.569
No	359 (92.3%)	214 (93.9%)	
Yes	30 (7.7%)	14 (6.1%)	
Lymph node metastasis			0.806
No	64 (16.5%)	35 (15.4%)	
Yes	325 (83.5%)	193 (84.6%)	
Lung metastasis			0.102
No	361 (92.8%)	202 (88.6%)	
Yes	28 (7.2%)	26 (11.4%)	
Peritoneal metastasis			<0.001
No	284 (73%)	202 (88.6%)	
Yes	105 (27%)	26 (11.4%)	

Ascites			<0.001
No	304 (78.1%)	208 (91.2%)	
Yes	85 (21.9%)	20 (8.8%)	
Other metastases			0.075
No	309 (79.4%)	195 (85.5%)	
Yes	80 (20.6%)	33 (14.5%)	
Number of metastatic sites			<0.001
0-1	211 (54.2%)	22 (9.6%)	
2	98 (25.2%)	135 (59.2%)	
≥3	80 (20.6%)	71 (31.1%)	
